# Preclinical investigation of artesunate as a therapeutic agent for hepatocellular carcinoma via impairment of glucosylceramidase-mediated autophagic degradation

**DOI:** 10.1038/s12276-022-00780-6

**Published:** 2022-09-20

**Authors:** Wenjia Chen, Zhaochen Ma, Lingxiang Yu, Xia MAO, Nan Ma, Xiaodong Guo, Xiaoli Yin, Funeng Jiang, Qian Wang, Jigang Wang, Mingliang Fang, Na Lin, Yanqiong Zhang

**Affiliations:** 1 Institute of Chinese Materia Medica, China Academy of Chinese Medical Sciences, Beijing, 100700 China; 2 The Fifth Medical Centre, Chinese PLA General Hospital, Beijing, 100039 China; 3 Artemisinin Research Center, China Academy of Chinese Medical Sciences, 100700 Beijing, China; 4 College of Life Science, South China Normal University, Guangzhou, 510631 China; 5 Guangdong Key Laboratory of Clinical Molecular Medicine and Diagnostics, South China University of Technology, Guangzhou, 510631 China; 6 State Key Laboratory of Natural and Biomimetic Drugs, School of Pharmaceutical Sciences, Peking University, Beijing, 100191 China; 7 Nanyang Technology University of Singapore, APT11-04 Singapore, Singapore

**Keywords:** Oncogenes, Drug development, Prognostic markers, Tumour biomarkers

## Abstract

Artesunate (ART) has been indicated as a candidate drug for hepatocellular carcinoma (HCC). Glucosylceramidase (GBA) is required for autophagic degradation. Whether ART regulates autophagic flux by targeting GBA in HCC remains to be defined. Herein, our data demonstrated that the dramatic overexpression of GBA was significantly associated with aggressive progression and short overall survival times in HCC. Subsequent experiments revealed an association between autophagic activity and GBA expression in clinical HCC samples, tumor tissues from a rat model of inflammation-induced HCC and an orthotopic mouse model, and human HCC cell lines. Interestingly, probe labeling identified GBA as an ART target, which was further verified by both a glutathione-S-transferase pulldown assay and surface plasmon resonance analysis. The elevated protein expression of LC3B, the increased numbers of GFP-LC3B puncta and double-membrane vacuoles, and the enhanced expression of SQSTM1/p62 indicated that the degradation of autophagosomes in HCC cells was inhibited by ART treatment. Both the in vitro and in vivo data revealed that autophagosome accumulation through targeting of GBA was responsible for the anti-HCC effects of ART. In summary, this preclinical study identified GBA as one of the direct targets of ART, which may have promising potential to inhibit lysosomal autophagy for HCC therapy.

## Liver cancer: Anti-enzyme potential drug candidate

Confirmation that the malaria drug artesunate targets a key enzyme overexpressed in aggressive liver cancer suggests it may be a novel therapeutic option for the disease. High levels of an enzyme called glucosylceramidase (GBA) are associated with poor prognosis in liver cancer, according to research conducted by Yanqiong Zhang and Na Lin at the Institute of Chinese Materia Medica, China Academy of Chinese Medical Sciences, Beijing, and co-workers. In experiments on rat and mouse models and human cell lines, the team demonstrated that high GBA levels over activated autophagic flux, accelerated the rate at which cellular material may be degraded and recycled in balanced, healthy cells. This disturbance enables liver cancer to progress. The researchers found that artesunate can suppress GBA expression levels and restore normal autophagic flux, boosting the drug’s anticancer activity.

## Introduction

Although surgical resection, ablation^1^, transplantation and systemic therapy have been authorized as therapeutic options for hepatocellular carcinoma (HCC)^2^, the prognosis of HCC is unfavorable, and the median overall survival time is less than three months^3^, indicating that more efficient therapeutic strategies are still needed to improve the clinical outcome of HCC patients. Autophagy is a type of programmed cell death occurring in all eukaryotic cells^4^. The entire autophagic process, called autophagic flux, contains a series of steps, including the formation of double-membrane vesicles (named autophagosomes) that engulf large intracellular components, the fusion of autophagosomes with lysosomes (to form autolysosomes) and the degradation of the contents, which may be completed in series once the autophagic response is induced and maintains cellular homeostasis^5^. With its name meaning “self-eating”, this lysosome-dependent catabolic degradation process has been indicated to be indispensable for cancer cell survival due to their high self-renewal capacity and blockade of autophagic flux^6^. Since autophagy has been found to exert either tumor-suppressive or oncogenic effects in various cancer types, its roles in malignant development and progression are still controversial. In particular, accumulating studies have indicated that the role of autophagy in HCC may be closely linked with the pathogenesis and developmental stage of this malignancy^7–10^. Hence, the identification of molecular targets and the investigation of the underlying mechanisms that modulate autophagy should attract more attention because of the great significance of these targets and mechanisms in the development and improvement of therapeutic strategies for HCC.

Glucosylceramidase (GBA), also called beta-glucosidase and glucocerebrosidase, is a lysosomal enzyme responsible for converting glycosylceramide to glucose and ceramide by cleaving the glycosidic linkage^11^. GBA loss-of-function mutations have been indicated to cause Gaucher disease and to be one of the major genetic risk factors for developing Parkinson’s disease due to the dysregulation of GBA-mediated autophagy^12^. Both hyperactivation and inactivation of GBA may change subcellular ceramide levels, which leads to disruption of autophagic flux^13^. Active GBA has been found to be associated with various human cancers, such as breast cancer^14^, gastric cancer^15^, HCC^16^ and prostate cancer^17^ in addition to neurodegenerative disorders. Notably, GBA overexpression and activation have been observed in primary HCC tissues and multiple HCC cell lines compared to their normal counterparts^16^. However, the clinical significance of GBA in HCC and the underlying mechanisms have not been thoroughly studied.

Artesunate (ART), a semisynthetic water-soluble derivative of artemisinin extracted from *Artemisia annua* L., has been extensively used as one of the first-line drugs for malaria therapy^18^. Given the satisfying clinical efficacy and safety of artemisinin and its derivatives in the treatment of malaria, it may be of great interest to repurpose them as potential anticancer drugs^19^. Accumulating studies providing both in vitro and in vivo evidence have revealed that ART may exert suppressive effects against a variety of human malignancies, including HCC, by regulating multiple pathways associated with cancer cell growth and proliferation, cell movement, cell survival and apoptosis, fatty acid metabolism, free radical scavenging and energy metabolism^20^. However, there is very little available information about how the regulatory effects of ART on autophagic flux that are involved in the development and progression of HCC. Thus, in-depth investigation of the mechanisms using clinical data and in vitro and in vivo models is needed to better understand the nature of the autophagy-related targets through which ART exerts its anti-HCC effects.

To determine the role of GBA in HCC and whether ART regulates autophagic flux by targeting GBA in HCC cells, we first validated the differential expression patterns of the GBA gene and protein in clinical HCC tissues and cell lines using microarray analysis, real-time quantitative PCR and immunohistochemistry. Then, the associations of GBA expression with HCC patients’ clinicopathological characteristics and prognosis were statistically evaluated based on both our clinical cohort and the TCGA dataset. Subsequent loss- and gain-of-function experiments were performed in vitro and in vivo to verify the oncogenic functions of GBA. After that, the interaction between ART and GBA was demonstrated by ART probe labeling, a glutathione-S-transferase (GST) pulldown assay and surface plasmon resonance (SPR) analysis. Moreover, both a rat model of diethylnitrosamine (DEN)-induced HCC and an orthotopic mouse model were established to evaluate the anti-HCC efficacy of ART. The pharmacological mechanisms of ART and its influence on autophagy in HCC cells by targeting GBA were further investigated.

## Materials and methods

### Ethics statement

This study was approved by the Research Ethics Committee of the Institute of Chinese Materia Medica and the Fifth Medical Centre of Chinese PLA General Hospital (license no: 2016003D). Animal experiments were carried out according to the guidelines for the care and use of laboratory animals of the Animal Ethical and Welfare Committee, Guangzhou Forevergen Medical Laboratory Animal Center, Guangdong, China (license no: IACUC-AEWC-F2005003).

### Human HCC samples

In our clinical cohort, a total of 99 cancerous and 18 adjacent noncancerous liver tissue samples from 100 HCC patients were collected at the Fifth Medical Centre of Chinese PLA General Hospital. Among these samples, three paired human primary HCC tissues and adjacent noncancerous liver tissues were used for microarray analysis, and the other tissues were used for experimental validation of GBA expression and its associations with various clinicopathological characteristics. To evaluate the prognostic value of GBA expression, a publicly available HCC dataset [Provisional, Tumor Samples with mRNA data (RNA Seq V2) (373 samples)] with mRNA sequencing expression data and relevant clinical information was obtained from The Cancer Genome Atlas (TCGA). Detailed information on our clinical cohort and the TCGA cohort is provided in Supplementary file-Section 1, Supplementary Tables 2 and 3.

### Cell culture and treatment

Human HCC cell lines (HepG2 and MHCC-97H) were purchased from American Type Culture Collection (ATCC; Rockville, MD, USA). Cells were maintained in Dulbecco’s modified Eagle’s medium (DMEM; HyClone, Logan, UT, USA) supplemented with 10% fetal bovine serum (FBS) and 1% penicillin G and streptomycin at 37 °C in a humidified atmosphere containing 5% CO_2_.

HepG2 cells were transiently transfected with small interfering ribonucleic acid (siRNA) constructs specific for GBA to knockdown the expression of GBA. The siRNA targeting sequences are provided in Supplementary file-Section 2. Transient transfection of HepG2 and MHCC-97H cells was performed using polyethylenimine (408727, Sigma–Aldrich, Germany). ART (S24000, shyuanye, Shanghai, China; IC_50_ of ART in HepG2 cells: 38.38 μM, IC_50_ of ART in MHCC-97H cells: 171.4 mM; all *P* *<* 0.05, Supplementary Fig. 1b) was used at 0.25, 0.50 and 1.00 IC_50_ in experiments to investigate its anti-HCC efficacy in vitro. ART was used at 0.50 IC_50_ to investigate the molecular mechanism by which it suppresses HCC. LTI-291 (10 nM, S1024, Selleck, Shanghai, China) was used to induce GBA expression in HepG2 cells. Bafilomycin A1 (BAF; 10 nM, S1413, Selleck, Shanghai, China) was used to inhibit late-stage autophagy in HepG2 cells.

### Rat model of inflammation-induced HCC

Male Sprague–Dawley (SD) rats (*n* = 80, 240–260 g in weight) were purchased from Guangdong Medical Laboratory Animal Center (production license no: SCXK 2013-0002, Guangzhou, China). All rats were housed under specific pathogen-free conditions in a room at a constant temperature of 24 °C ± 1 °C on a 12-h light/12-h dark cycle and were provided access to water and food *ad libitum*. Prior to the experiments, the rats were allowed a one-week acclimatization period.

The rats were randomly divided into four groups: normal control (*n* = 15), normal+ART (*n* = 15), DEN model (*n* = 25) and DEN + ART (*n* = 25). Rats in the normal control group were fed a normal diet, rats in the normal-ART group received daily oral administration of 28.8 mg/kg ART (S24000, shyuanye, Shanghai, China) with normal drinking water, and rats in both the DEN model and DEN + ART groups were given 0.01% DEN (N0756, Sigma–Aldrich, USA) ad libitum in the drinking water for a period of 18 weeks. In addition, rats in the DEN + ART group received daily oral administration of 28.8 mg/kg ART beginning on the day of DEN modeling for a period of 18 weeks. The dose selected for ART (28.8 mg/kg) was twice the daily clinical dose used for hepatic fibrosis patients in clinics, which has been indicated to achieve the highest therapeutic efficacy in patients with hepatic fibrosis.

The rat body weight, liver index and ratio of liver weight to body weight were measured as previously described^21^. The livers collected from different groups were sectioned and fixed with 10% formalin. The remaining portions of the livers were snap-frozen and stored at −80 °C until use.

### Orthotopic tumor model in nude mice

Sixty-four BALB/c nude mice (weight: 15 ± 2 g, age: 7–8 weeks) were purchased from Guangdong Medical Laboratory Animal Center, Guangzhou, China (license no: SCXK-2018-0186) and were housed five per cage in wire-top cages with sawdust bedding in an isolated, clean, air-conditioned room at a temperature of 25–26 °C and a relative humidity of ~50%. The light was on for 12 h/day. An orthotopic mouse model of HCC was established as previously described^22^. After sterilization of the mouse abdomen with 70% ethanol, a midline incision was made through the skin and peritoneal membrane beginning 1.5 cm from the xiphoid process and continuing over the abdomen. The right medial lobe of the liver was removed from the abdominal cavity and injected with a clonal population of HepG2 cells or HepG2 + LTI-291 cells (3 × 10^6^ cells per mouse), and a drop of OB anastomotic glue was applied at the incision to seal the incision. On the eighth day postsurgery, the mice were divided into the control, HepG2, HepG2 with various doses of ART (low: 5 mg/kg, moderate: 10 mg/kg, high: 20 mg/kg), HepG2 + LTI-291, and HepG2 + LTI-291 with various doses of ART (low: 5 mg/kg, moderate: 10 mg/kg, high: 20 mg/kg) groups. The corresponding formulations were administered intraperitoneally once every two days for six weeks. Seven weeks after administration, mice were sacrificed by cervical dislocation, and tumor tissues were collected for subsequent analysis.

### Gene expression profiling

The mRNA expression profiles in HCC and adjacent noncancerous liver tissue samples were obtained using an Affymetrix Clariom D Human array; analysis was carried out by Shanghai GMINIX Biotechnology Corporation (Shanghai, China). The data are accessible through the NCBI Gene Expression Omnibus under accession number GSE166163. Detailed information on gene expression profiling is provided in Supplementary file-Section 3.

### Real-time quantitative PCR analysis

GBA mRNA expression levels in clinical sample tissues and HCC cells were examined by quantitative PCR using an ABI 7900HT quantitative PCR system. GAPDH was used as the internal control for normalization and quantification. The primer sequences are provided in Supplementary file-Section 4. Relative quantification of GBA mRNA expression was performed using the comparative cycle threshold method.

### Immunohistochemical & Immunofluorescence analyses

Immunohistochemical staining and immunofluorescence analyses were carried out according to our previous description^23^. The detailed protocols are provided in Supplementary file-Section 5.

### Cell proliferation, cell cycle, apoptosis & colony formation assays

To evaluate the malignant behaviors of HepG2 and MHCC-97H cells in different groups, CCK-8, cell cycle, apoptosis and colony formation assays were carried out according to the protocols described in our previous studies^23,24^. The detailed protocols are provided in Supplementary file-Section 6.

### Western blot analysis

The protein expression levels of GBA, LC3, and p62 in clinical tissue samples and HCC cells in different groups were determined by Western blot analysis according to the protocol described in our previous studies^23,24^. Detailed information on the primary antibodies is provided in Supplementary file-Section 7.

### Transmission electron microscopy and evaluation of autophagosomes

Transmission electron microscopy was used to visualize autophagosomes in HCC cells and orthotopic HCC tissues that were fixed with a glutaraldehyde (2.5%)/paraformaldehyde (1%) mixture for 1 h at 4 °C in 100 mM phosphate buffer (pH 7.2). The samples were postfixed with 1% osmium tetroxide for 2 h at 4 °C, dehydrated in a graded acetone series, embedded in Araldite (Fluka, 10951), sectioned, double stained with uranyl acetate and lead citrate, and analyzed using a transmission electron microscope (JEOL, JEM1230). Autophagy was quantified by calculating the average number of autophagosomes; autophagosomes were counted and averaged in five or more cells per group according to a previous description^25,26^.

### Pulldown/LC–MS/MS analysis and target validation

To identify the cellular interaction targets of ART, pulldown (PD) assays followed by Western blotting and LC–MS/MS were performed. Detailed information on the protocols is provided in Supplementary file-Section 8.

### Surface plasmon resonance (SPR) analysis

The binding kinetics between GBA and ART were evaluated by SPR analysis using a Biacore T200 instrument (GE Healthcare, Pittsburgh, PA, USA) according to the protocol described in our previous studies^27–29^. The dissociation constant (K_D_) was calculated according to BIAevaluation software (version 4.1, GE Healthcare, Pittsburgh, PA, USA).

### Detection of biochemical indicators and scoring of fibrosis-related indices

Serum levels of alanine aminotransferase (ALT), aspartate aminotransferase (AST), alkaline phosphatase (ALP) and the liver fibrosis marker HA in the different groups were determined using an automatic analyzer (AU5831; Beckman Coulter, CA, USA). In addition, serum levels of AFP in the different groups were measured using an ELISA kit [cat. no. CD-010616R, Rat AFP ELISA Kit, Wuhan Purity Biological Technology Co., Ltd, Wuhan, China (http://chundubio.com/)] according to the manufacturer’s instructions.

### Histopathological evaluation

To evaluate the pathological changes in liver tissues in different groups of rats with inflammation-induced HCC, the excised livers were fixed with 10% formalin. Following routine procedures, 3-μm-thick sections were sliced from the resulting paraffin blocks. After deparaffinization and rehydration, the sections were stained with hematoxylin and eosin. All sections were examined under a light microscope (ML31, Mshot, Guangzhou, China).

### Statistical analyses

All experiments in the current study were performed in triplicate. Data are shown as the mean ± SD values, and statistical analyses were performed using GraphPad Prism (version 8.0, GraphPad Software, CA, USA). *P* values <0.05 were considered to be significant. Unpaired Student’s *t* test was used for comparisons between two groups where appropriate. To evaluate the clinical implications, the HCC patients in the TCGA dataset were divided into the high GBA expression and low GBA expression groups using the median value of GBA expression. Associations between GBA expression and various clinicopathological characteristics of HCC patients were evaluated by Fisher’s exact test for 2 × 2 contingency tables and Pearson *χ*^2^ test for non-2 × 2 contingency tables. The Kaplan–Meier method with the log-rank test was used for survival analysis.

## Results

### GBA overexpression confers poor prognosis on HCC patients and is associated with impaired autophagic degradation in human HCC tissues

**Fig. 1:**
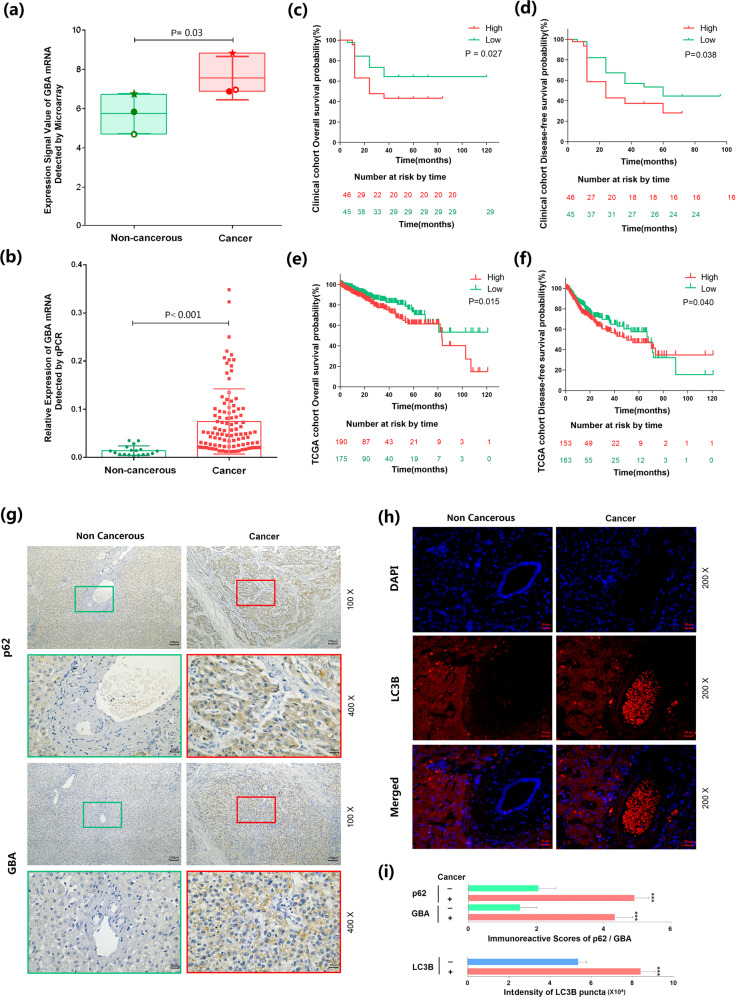
GBA overexpression is associated with poor prognosis in HCC patients and impaired autophagic degradation in human HCC tissues. **a** Signal values indicating GBA mRNA expression detected by analysis of a microarray containing three pairs of noncancerous and cancerous liver tissues collected from three HCC patients (cancerous vs. noncancerous, *P* = 0.03). **b** Expression levels of GBA mRNA detected by quantitative real-time PCR using 99 HCC and 18 noncancerous liver tissues (cancerous vs. noncancerous, *P* < 0.001). Each dot represents information from one patient. The data are presented as the mean ± SEM values; *p* values were calculated by two-tailed unpaired *t* test. **c,** **d** Kaplan–Meier curves of overall (*P* = 0.027) and disease-free (*P* = 0.038) survival of HCC patients stratified according to the GBA mRNA expression level based on our clinical cohort; **e**, **f** Kaplan–Meier curves of overall (*P* = 0.015) and disease-free (*P* = 0.040) survival of HCC patients based on the TCGA dataset, respectively. The data are presented as proportions; *p* values were calculated by the log-rank test. **g** Immunohistochemical analysis of the GBA and SQSTM1/p62 protein expression levels in clinical HCC and noncancerous liver tissues. Original magnifications, ×100 and ×400; scale bars, 100 μm and 25 μm. **h** Immunofluorescence analysis of the subcellular localization and expression level of LC3B protein in clinical HCC and noncancerous liver tissues. Original magnification, ×200; scale bar, 50 μm. **i** Semiquantitative results of the immunoreactive scores of GBA and SQSTM1/p62 proteins and the intensity of LC3B puncta in clinical HCC and noncancerous liver tissues. *p < 0.05, **p < 0.01 and ***p < 0.001 compared with the noncancerous liver tissues.

The microarray data in Fig. 1a show that the level of GBA mRNA was significantly higher in human HCC tissues than in noncancerous liver tissues (cancerous vs. noncancerous, 7.54 ± 1.10 vs. 5.73 ± 1.02, *P* = 0.03), and this result was validated by real-time quantitative PCR using our clinical cohorts (cancerous vs. noncancerous, 0.07 ± 0.07 vs. 0.01 ± 0.01, *P* < 0.001; Fig. 1b). To evaluate the clinical significance of GBA in human HCC, the median value (0.506) of GBA expression in all 99 human HCC tissues was used to divide the patients into the high and low GBA expression groups (n = 50 and 49, respectively). Statistical analysis showed that more HCC patients in the high GBA expression group than in the low GBA expression group showed high preoperative serum AFP levels (0.01 < *P* < 0.05) and high tumor grades (P < 0.01, Supplementary Table 1). Similarly, the statistical results based on the TCGA dataset revealed that dramatic overexpression of GBA was significantly associated with high age (*P* = 0.02), the presence of metastasis (0.01 < *P* < 0.05) and vascular invasion (*P* = 0.04), and a short overall survival time (*P* = 0.01) in HCC patients (Supplementary Table 2). In addition, Kaplan–Meier survival analysis based on both our clinical cohort and the TCGA cohort indicated that HCC patients with high GBA expression had worse overall (*P* = 0.027 and *P* = 0.015; Fig. 1c, e, respectively) and disease-free (*P* = 0.038 and *P* = 0.040, Fig. 1d, f, respectively) survival rates than those with low expression. Moreover, compared with the adjacent noncancerous liver tissues, the representative human HCC samples from the same patients displayed strong positive staining for the GBA protein and SQSTM1/p62 accumulation (Fig. 1g), as well as an increased number of GFP-LC3B puncta (Fig. 1h). The quantitative data indicating statistical significance are shown (all *P* < 0.001, Fig. 1i).

### GBA functions as an oncogene in HCC

**Fig. 2:**
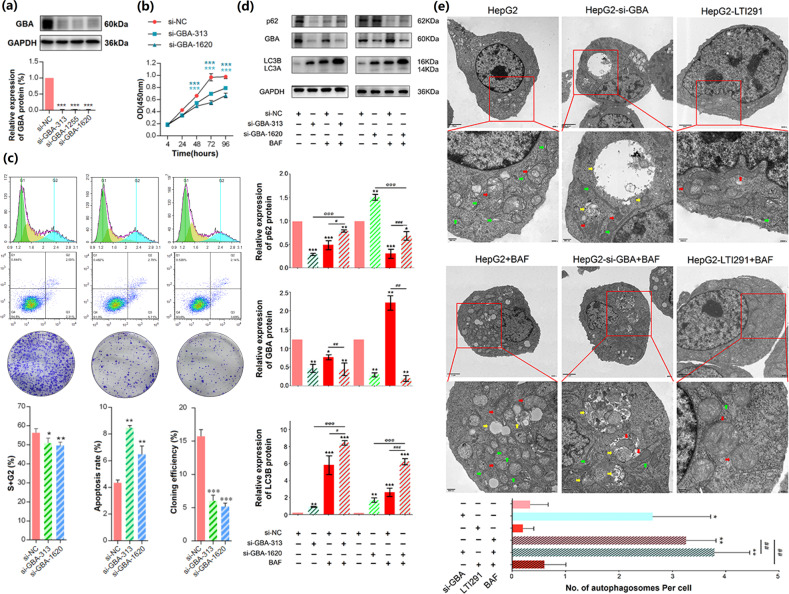
Effects of GBA on cell malignant behaviors and associations between GBA and autophagy-related protein (SQSTM1/p62 and LC3B) expression in HepG2 cells. **a** Protein expression levels of GBA in HepG2 cells transfected with three si-GBA plasmids (si-GBA-313, si-GBA-1250 and si-GBA-1620), as determined by western blot analysis. Representative column charts showing relative protein expression. **b** Viability of HepG2 cells transfected with si-GBA-313 and si-GBA-1620, as determined by a CCK-8 assay. **c** Flow cytometric, apoptosis and cloning efficiency analyses of the cell cycle distribution of HepG2 cells transfected with si-GBA-313 and si-GBA-1620. The data are expressed as the mean ± SD values. *p < 0.05, **p < 0.01, ***p < 0.001; si-GBA groups compared with the si-NC group. **d** Protein expression levels of GBA, SQSTM1/p62 and LC3B in HepG2 cells of different groups (BAF: 10 nM), as determined by western blot analysis. GAPDH was used as the internal control. **e** TEM images of different groups of cells (LTI-291: 10 nM, BAF: 10 nM). The red arrows indicate mitochondria, the green arrows indicate rough endoplasmic reticulum, and the yellow arrows indicate autophagosomes. Original magnifications, ×8000 and ×20,000; scale bars, 2 μm and 500 nm. The data are expressed as the mean ± SD values. *p < 0.05, **p < 0.01, and ***p < 0.001 compared with the si-NC group; ^#^*p* < 0.05, ^##^*p* < 0.01, and ^###^*p* < 0.001 compared with the si-NC-BAF group; ^@^*p* < 0.05, ^@@^*p* < 0.01, and ^@@@^*p* < 0.001 compared with the si-GBA group.

To investigate the function of GBA in HCC, two human HCC cell lines, HepG2 and MHCC-97H, which were demonstrated to have high GBA expression (Supplementary Fig. 1a), were transfected with three si-GBA plasmids (si-GBA-313, si-GBA-1255 and si-GBA-1620) to reduce GBA expression. Then, the western blot data demonstrated that the expression levels of GBA in all si-GBA transfection groups were markedly lower than those in the si-NC transfection group (all P *<* 0.05, Fig. 2a and Supplementary Fig. 2a). Measurement of the cell proliferation ability by a CCK-8 assay indicated that the OD values of both HepG2 and MHCC-97H cells transfected with the si-GBA plasmids were significantly lower than those transfected with the si-NC plasmid at different time points (HepG2 cells: after 48 h, MHCC-97H cells: after 24 h, all P *<* 0.05; Fig. 2b and Supplementary Fig. 2b). In addition, the knockdown of GBA effectively induced apoptosis in HCC cells by increasing the percentages of early-stage and late-stage apoptotic cells (both HepG2 and MHCC-97H cells, *P* *<* 0.01; Fig. 2c, Supplementary Fig. 2d and Supplementary Fig. 2g). Moreover, evaluation of cell viability by a colony formation assay showed that the more colonies formed in the si-NC transfection groups than in the si-GBA transfection groups (both HepG2 and MHCC-97H cells, *P* *<* 0.01; Fig. 2c, Supplementary Fig. 2e and Supplementary Fig. 2h). We also observed G1 arrest in both HepG2 and MHCC-97H cells when GBA expression was inhibited (all *P* *<* 0.05, Fig. 2c, Supplementary Fig. 2c and Supplementary Fig. 2f). In contrast, the inhibitory effects of GBA knockdown on HCC cell proliferation, cell cycle progression and cell colony formation and its inductive effect on apoptosis were significantly reversed by treatment with the GBA activator LTI-291 (all *P* *<* 0.05, Fig. 4a).

### GBA knockdown leads to impaired autophagic degradation in HCC cells

It is worth noting that the protein expression levels of LC3B and SQSTM1/p62 were significantly increased in HCC cells with si-GBA transfection, and these levels were further increased by combination treatment with si-GBA transfection and the late-stage autophagy inhibitor BAF compared to those in cells with si-GBA transfection or BAF treatment alone (LC3B protein, all *P* *<* 0.05 when using two si-GBA plasmids; SQSTM1/p62 protein, all *P* *<* 0.05 when using the si-GBA-313 plasmid, Fig. 2d). In addition, transmission electron microscopy was used to observe the ultramicrostructure of HCC cells in the different treatment groups. Remarkably, numerous double- or multimembrane autophagosomes were found in GBA knockdown cells, and the number of these structures was increased by combination treatment with si-GBA transfection and BAF (all *P* *<* 0.01, Fig. 2e). In contrast, the number of autophagosomes in HCC cells treated with the GBA activator LTI-291 was lower than that in HepG2 cells with or without si-GBA transfection (Fig. 2e). Notably, combination treatment with LTI-291 and BAF significantly reversed the increase in autophagosome accumulation induced by BAF alone (*P* *<* 0.01, Fig. 2e). Consistent with these findings, reduced protein expression levels of LC3B and SQSTM1/p62 were found in HCC tissues from tumor-bearing nude mice with GBA activation by LTI-291 treatment (Fig. 7), which was in line with the observations made by transmission electron microscopy (Fig. 6). These findings suggest that GBA knockdown may result in impaired autophagic degradation in HCC cells, which was partially reversed by activation of GBA.

### GBA is a direct target of ART

**Fig. 3:**
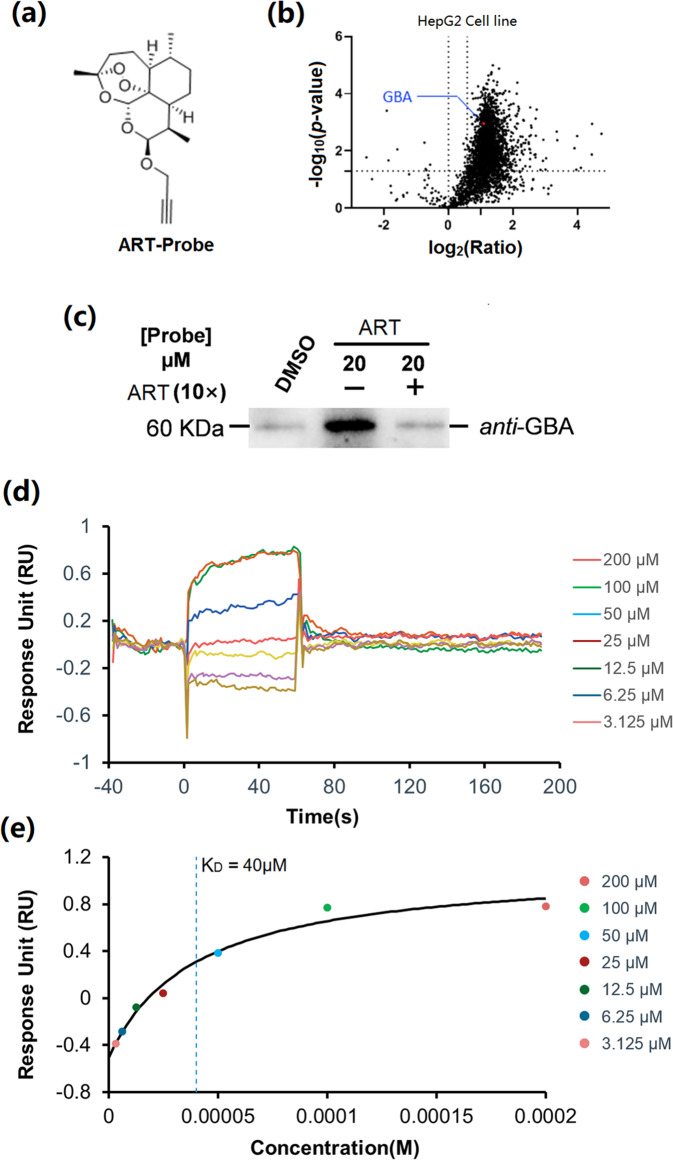
GBA is the direct target of ART. **a** Chemical structure of the ART probe. **b** Mass spectrometry-based profiling of the ART probe (20 μM) in the presence of excess ART (10×). **c** Pulldown/western blot results for target validation of the ART probe (20 μM). **d** BIAcore surface plasmon resonance (SPR) kinetic analyses of ART binding to GBA. Sensorgram and saturation curve of the titration of ART on GBA immobilized on a CM5 chip. **e** Binding curves of ART to GBA were fit to a steady-state affinity model to obtain the *K*_D_ value.

The ART probe (Fig. 3a), with a minimalist alkyne-containing linker incorporated into ART^29^, was employed to identify cellular targets of ART by large-scale chemoproteomic experiments. HepG2 cells were treated with the ART probe. Proteins in the cell lysates were conjugated to biotin-N3, and the resulting probe-labeled proteins were affinity-purified and identified by LC–MS/MS. Control experiments with the probes in the presence of the corresponding parent competitor (ART) were carried out concurrently, and the results were used to distinguish between real targets and background labeling. The identified protein hits were analyzed on corresponding volcano plots showing the -log2 of the competition ratio (probe/probe with excess competitor) plotted against the statistical significance (−log10 *p* value). Proteins with a p value less than 0.05 and a competition ratio greater than 1.5 were considered to be significant hits. As expected, GBA was successfully identified as one of the direct targets of ART (Fig. 3b) and further validated by a pulldown assay followed by WB (Fig. 3c), which proved the direct binding of ART to GBA. Consistent with the above findings, the SPR data showed that ART bound strongly to GBA with a *K*_D_ value of 40 μM (Fig. 3d, e).

### ART suppresses HCC cell malignancy by targeting GBA

To explore the effects of ART on HCC cell malignancy, the cytotoxicity of ART was evaluated in HepG2 and MHCC-97H cells by a CCK-8 assay. As shown in Supplementary Fig. 3a, e, ART induced cytotoxicity in these two HCC cell lines in a dose-dependent manner and was also confirmed to reduce the number of cell colonies in the colony formation assay (all P *<* 0.05, Supplementary Fig. 3d for HepG2 cells; Supplementary Fig. 3h for MHCC-97H cells). In addition, G1 arrest was observed in both HepG2 and MHCC-97H cells treated with various concentrations of ART (all *P* *<* 0.05, Supplementary Fig. 3b for HepG2 cells; Supplementary Fig. 3f for MHCC-97H cells). We also found that ART increased the apoptosis rate of these two HCC cell lines in a dose-dependent manner (all *P* *<* 0.05, Supplementary Fig. 3c for HepG2 cells; Supplementary Fig. 3g for MHCC-97H cells). All these effects were similar to the influence of GBA knockdown in HCC cells, as shown in Fig. 2 and Supplementary Fig. 2. The above data indicate that HepG2 cells were more sensitive than MHCC-97H cells to ART. Further study was conducted mainly in HepG2 cells.

**Fig. 4:**
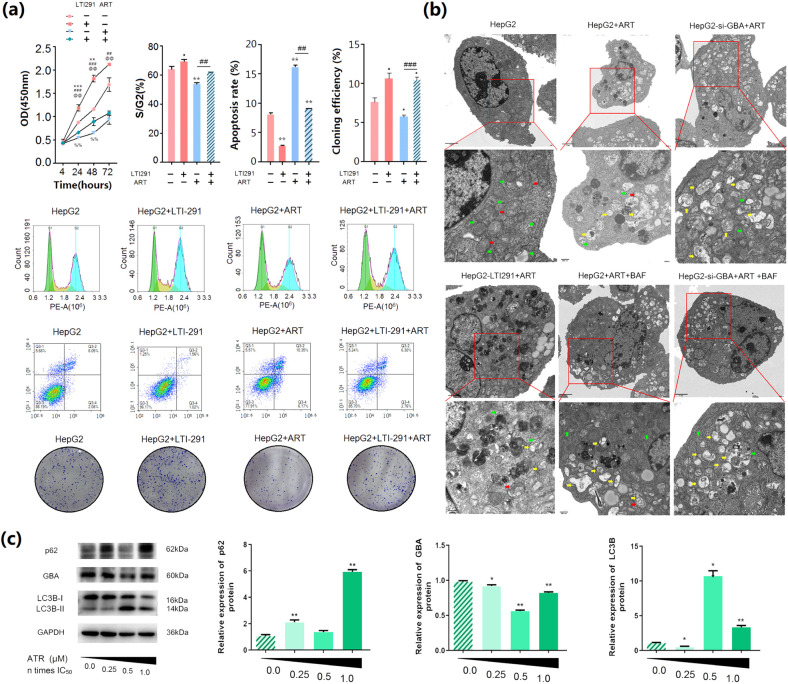
Effects of the GBA activator LTI-291 on the malignant behaviors of HepG2 cells and the regulatory effects of ART on the expression of autophagy-related proteins (GBA, SQSTM1/p62 and LC3B) and the number of autophagosomes in HCC cells. **a** Proliferation of HepG2 cells treated with the GBA activator LTI-291 (10 nM) alone or in combination with ART (20 μM, 0.5 IC_50_), as evaluated using a CCK-8 assay. Apoptosis of HepG2 cells treated with the GBA activator LTI-291 (10 nM) alone or in combination with ART (20 μM, 0.5 IC_50_), as determined by a TUNEL assay. Flow cytometric analysis of the effects of LTI-291 treatment alone or in combination with ART on the cell cycle distribution of HepG2 cells. Colony formation ability of HepG2 cells treated with the GBA activator LTI-291 alone or in combination with ART, as evaluated by a colony formation assay. The data are expressed as the mean ± SD values. *p < 0.05, **p < 0.01, and ***p < 0.001 compared with the blank control group; ^#^*p* < 0.05, ^##^*p* < 0.01, and ^###^*p* < 0.001 compared with the LTI-291 monotherapy group; ^@^*p* < 0.05, ^@@^*p* < 0.01, and ^@@@^*p* < 0.001 compared with the ART monotherapy group. (**b**) Transmission electron microscopy images of HepG2 cells treated with ART at different concentrations (ART: 20 μM, 0.5 IC50; LTI-291: 10 nM; BAF: 10 nM). The red arrows indicate mitochondria, the green arrows indicate endoplasmic reticulum, and the yellow arrows indicate autophagosomes. Original magnifications, ×8000 and ×20,000; scale bars, 2 μm and 500 nm. (**c**) Protein expression levels of GBA, SQSTM1/p62 and LC3B in HCC cells treated with ART at different concentrations (40 μM, IC_50_), as determined by western blot analysis. GAPDH was used as the internal control. The data are expressed as the mean ± SD values. *p < 0.05, **p < 0.01, and ***p < 0.001 compared with the blank control HepG2 cell group.

To further determine whether GBA might be responsible for the inhibitory effects of ART in HCC cell malignancy, the GBA activator LTI-291 (10 nM) was used in combination with ART (20 μM, 0.5 IC50). The inhibitory effects of ART on HCC cell proliferation, cell cycle progression and cell colony formation, as well as its inductive effect on apoptosis, were reversed by combined treatment with LTI-291 (10 nM) (all *P* *<* 0.05, Fig. 4a).

### ART impairs autophagic degradation of HCC cells by targeting GBA

ART promoted the conversion of LC3B-I to LC3B-II and elevated the protein expression of SQSTM1/p62 but reduced the protein expression of GBA in HCC cells (all *P* *<* 0.05, Fig. 4c). Interestingly, ART treatment significantly increased the number of autophagosomes in HCC cells, and this increase was enhanced by combined treatment with si-GBA transfection but reversed by the combined treatment with LT1-291 (all *P* *<* 0.05, Fig. 4b), as evaluated by transmission electron microscopy. To determine whether the accumulation of autophagosomes induced by ART treatment in HCC cells was due to impaired degradation, we measured autophagic flux with BAF, which is an inhibitor of late-stage autophagy, and found that the number of autophagosomes in HCC cells treated with the combination of BAF and ART or the combination of BAF, ART and si-GBA transfection was greater than that in cells treated with ART alone (both *P* *<* 0.05), Fig. 4b.

### ART attenuates inflammation-induced hepatic carcinogenesis by impairing GBA-mediated autophagic degradation

To verify the hypothesis that ART can attenuate inflammation-induced hepatic carcinogenesis, a stable rat model with DEN-induced progression of hepatic fibrosis to cirrhosis to cancer was established and used to assess the therapeutic effects of ART. No rats treated with ART (28.8 mg/kg) died, and DEN-induced injury in rats resulted in progressive liver fibrosis and cirrhosis followed by HCC over different time periods. In detail, repeated administration of DEN (0.01%) for 8 weeks caused fibrosis, and advanced fibrosis and cirrhosis occurred after 12 weeks. All rats in the model group had marked neoplastic lesions by 18 weeks of DEN administration. Multiple white nodules were observed on the liver surface in mice in the DEN model group, which were effectively attenuated by the administration of ART (Supplementary Fig. 4a). Histologically, we observed normal hepatic lobular architecture, normal hepatocytes and a sinusoidal architecture without fibroplasia and inflammatory cell infiltration but only scattered foci of fatty degeneration in normal control group and normal-ART group rats (marked with green arrows in Supplementary Fig. 4b). After administration of DEN for 8 weeks, slight microscopic fibrosis occurred and continued to develop into cirrhosis, as well as hemorrhagic necrosis with inflammatory cell foci around fibrotic tissue, at the 12th week (marked with red arrows in Supplementary Fig. 4b). Subsequently, the incidence of tumors was markedly increased. All the rats in the DEN model group developed HCC by week 18. The white nodules observed on the surface of the livers were histologically confirmed to be HCC; the cells in these nodules had clear, eosinophilic or hyperbasophilic cytoplasm, and all had enlarged and hyperchromatic nuclei (marked with blue arrows in Supplementary Fig. 4b). Interestingly, administration of ART effectively reversed the pathological changes in the liver by reducing the inflammatory level and malignancy degree at the indicated times (Supplementary Fig. 4b). These findings were in line with the above gross anatomical observations.

In addition, the body weights and liver indices of rats in the DEN model and DEN + ART treatment groups were significantly lower and higher, respectively, than those in the normal control and normal-ART groups at the indicated times (all *P* *<* 0.05, Supplementary Fig. 5a, b). There were no significant differences in body weights or liver indices between the normal control and normal-ART groups or between the DEN model and DEN + ART groups (Supplementary Fig. 5a, b).

Moreover, the serum levels of several liver function markers, such as ALT, AST and ALP, in the DEN model group were significantly higher than those in the normal control and normal-ART groups and remained high until week 18 (all *P* *<* 0.05, Supplementary Fig. 6a–c). After DEN administration was begun, the serum level of the fibrosis marker HA was high, with a statistically significant increase from baseline, from week 12 to week 18 (all *P* *<* 0.05, Supplementary Fig. 6d). A gradual increase in the tumor-related serum marker AFP was observed beginning in week 12, and its serum levels were significantly higher than those in the normal control and normal-ART groups (all *P* *<* 0.01, Supplementary Fig. 6e). Importantly, administration of ART effectively reduced the increasing serum levels of ALT, AST, ALP, HA and AFP induced by DEN administration (all *P* *<* 0.05, Supplementary Fig. 6a–e).

**Fig. 5:**
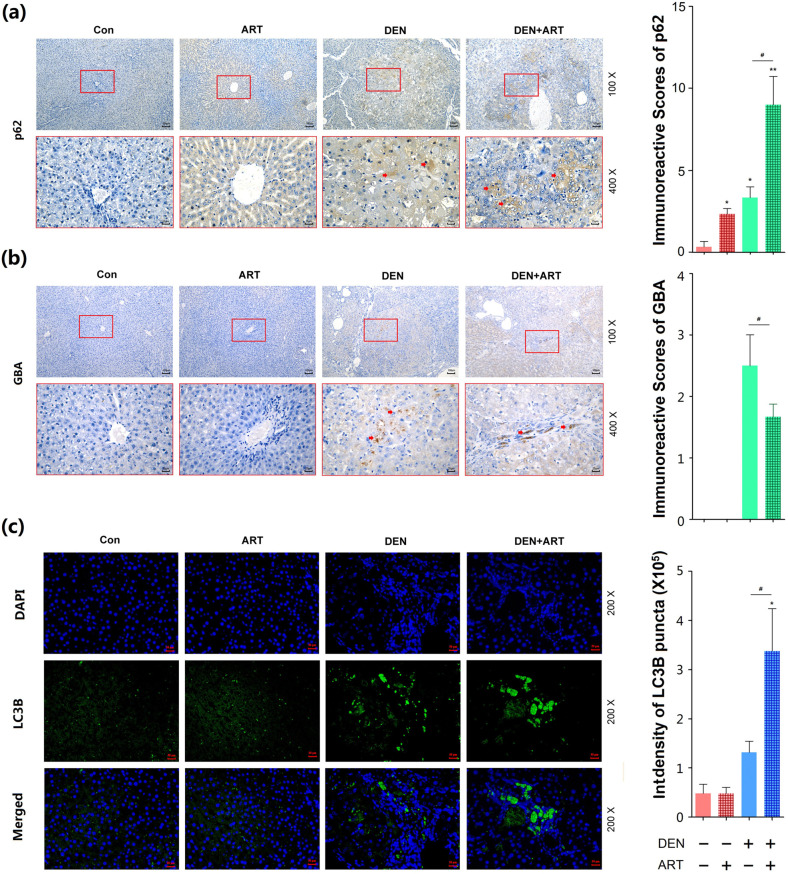
Regulatory effects of ART on the expression of autophagy-related proteins (GBA, SQSTM1/p62 and LC3B) in liver tissues obtained from rats with DEN-induced HCC. **a**, **b** Immunostaining and immunoreactive scores of GBA and SQSTM1/p62 proteins in liver tissues of rats with DEN-induced HCC that were treated with or without ART (20 μM, 0.5 IC_50_). Original magnifications, 100× and 400×; scale bars, 100 μm and 25 μm. **c** Immunofluorescence analysis of LC3B protein expression in liver tissues of rats with DEN-induced HCC that were treated with or without ART (20 μM, 0.5 IC_50_). Original magnification, 200×; scale bar, 50 μm. The data are expressed as the mean ± SD values. *p < 0.05, **p < 0.01, and ***p < 0.001 compared with the normal control group; ^#^*p* < 0.05, ^#^*p* < 0.01, and ^###^*p* < 0.001 compared with the DEN model group.

To determine whether the inhibitory effects of ART on inflammation-induced hepatic carcinogenesis were associated with GBA-mediated autophagic degradation, the expression levels and subcellular localization of GBA, SQSTM1/p62 and LC3B proteins in cancerous and noncancerous liver tissues of the different groups were assessed by immunohistochemical and immunofluorescence assays. Figure 5a, b shows the stronger positive cytoplasmic staining of GBA and SQSTM1/p62 proteins induced by DEN. Following administration of ART, the protein expression level of GBA was significantly decreased but that of SQSTM1/p62 was markedly enhanced (all *P* *<* 0.05, Fig. 5a, b). Consistent with these findings, our immunofluorescence assay showed a marked number of GFP-LC3B puncta in the cytoplasm of tumor cells in liver tissues after DEN administration, and the number of these puncta was also increased by ART (all *P* *<* 0.05, Fig. 5c).

### ART reduces tumor burden and impairs GBA-mediated autophagic degradation in orthotopic tumor-bearing mice

**Fig. 6:**
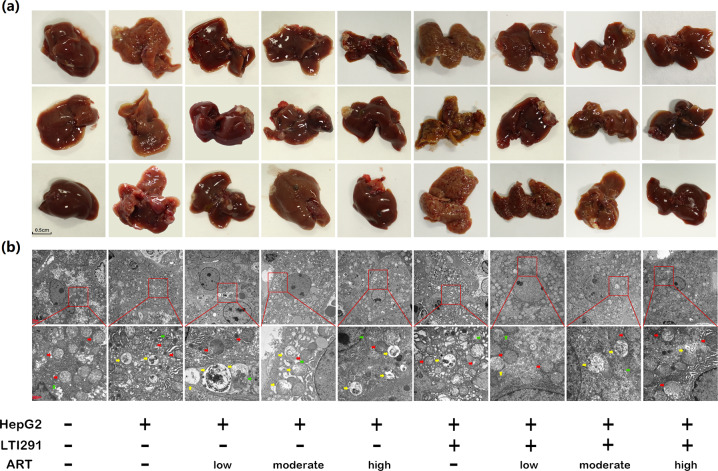
ART reduces tumor burden and impairs GBA-mediated autophagic degradation in orthotopic tumor-bearing mice. **a** Generation of neoplastic lesions in the different groups. **b** Transmission electron microscopy images of the different groups. The red arrows indicate mitochondria, the green arrows indicate rough endoplasmic reticulum, and the yellow arrows indicate autophagosomes. Original magnifications, ×8000 and ×20,000; scale bars, 2 μm and 500 nm.

**Fig. 7:**
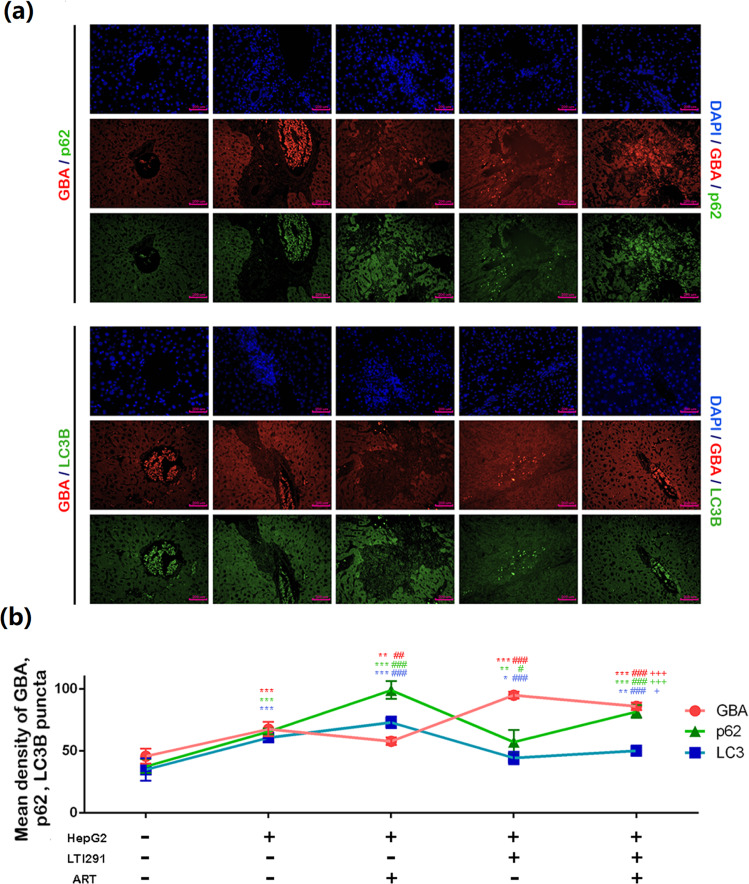
Regulatory effects of ART on the expression of autophagy-related proteins (GBA, SQSTM1/p62 and LC3B) in liver tissues obtained from orthotopic tumor-bearing mice. **a**, **b** Double immunofluorescence staining of GBA/p62 and GBA/LC3B proteins in liver tissues of orthotopic tumor-bearing mice in the different groups (ART: 20 μM, 0.5 IC50; LTI-291: 10 nM; BAF: 10 nM). Original magnification, ×200; scale bar, 100 μm. The data are expressed as the mean ± SD values. *p < 0.05, **p < 0.01, and ***p < 0.001 compared with the normal control group; ^#^p < 0.05, ^##^*p* < 0.01, and ^###^*p* < 0.001 compared with the HepG2 group; ^+^*p* < 0.05, ^++^*p* < 0.01, and ^+++^*p* < 0.001 compared with the HepG2 + LTI-291 group.

To determine the anti-HCC effect of ART in vivo, an orthotopic tumor model, which may naturally represent a native tumor because of the growth of tumor cells in the liver, was established in nude mice, and the mice were then treated with ART (low dose: 5 mg/kg, moderate dose: 10 mg/kg, high dose: 20 mg/kg). As shown in Fig. 6a, administration of ART effectively decreased the tumor size compared with that in the model group. Next, we examined the regulation of autophagy by ART in tumor samples. Consistent with the in vitro findings, administration of ART significantly enhanced autophagosome accumulation in tumor tissues according to transmission electron microscopy observation (*P* *<* 0.05, Fig. 6b). Interestingly, the number of autophagosomes in mice treated with the combination of ART and LTI-291 was decreased and increased compared to that in mice treated with ART or LTI-291 alone, respectively (Fig. 6b). We further revealed the colocalization of GBA with LC3 and SQSTM1/p62 proteins in tumor tissues from mice in the different groups by immunofluorescence assays. Notably, in tumor samples, the protein levels of LC3B and SQSTM1/p62 were increased but the level of GBA was reduced by ART (Fig. 7a, b), and both of these changes were reversed by LTI-291 (Fig. 7a, b), implying an association between the anticancer effects of ART and GBA-mediated autophagic degradation.

## Discussion

**Fig. 8:**
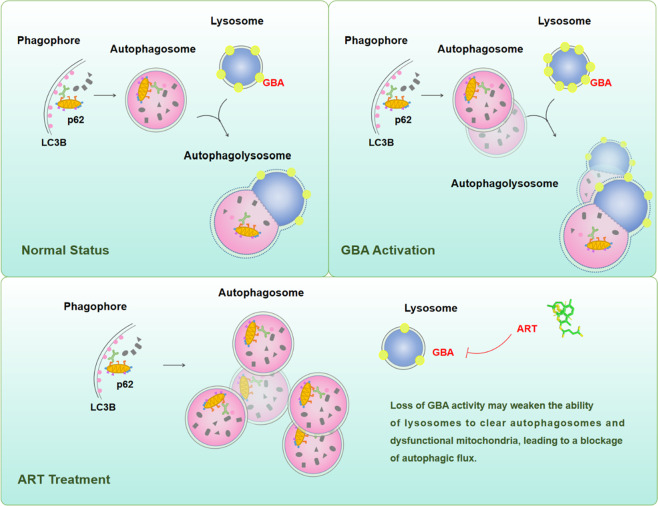
Illustration of the underlying mechanisms of GBA-mediated autophagic degradation in HCC progression and treatment. This schematic model illustrates the autophagosome formation when GBA is activated or suppressed following the treatment of ART versus the normal status of autophagic flux. Top left: GBA-encoded glucocerebrosidase (GCase) is a lysosomal hydrolase, which means that GBA mainly acts in the degradation of autophagosomes. Top right: The overexpression of GBA is closely related to the malignant progression and poor prognosis of HCC, suggesting that the activation of GBA may lead to autophagy of HCC cells and promote the cellular viability, thus promoting the malignant development of HCC. Bottom: ART inhibits the abnormally high expression of GBA and prevents the degradation of autophagosomes in HCC, leading to blocking the autophagic flux.

Accumulating preclinical and clinical evidence shows that the modulation of altered autophagic flux may be a promising anticancer therapeutic strategy^23,30^. In normal hepatocytes, basal autophagy has a housekeeping function that may be involved in maintaining liver homeostasis and preventing malignancy by removing damaged mitochondria and transformed liver cells^24^. In contrast, autophagy has been demonstrated to be activated or inhibited in different cancers, indicating that autophagy may play a dual role in inhibiting and promoting the survival of malignant cells^31^. The autophagic machinery is an attractive target for the discovery of novel anticancer agents. In the current study, we first observed GBA mRNA and protein overexpression in human HCC tissues compared with paired adjacent noncancerous liver tissues, and this finding was supported by the data obtained from analysis based on the TCGA database and by our in vitro and in vivo experimental data. This distinct expression status of GBA was indicated by statistical analysis to be associated with aggressive clinicopathological features in human HCC tissues and to be a predictive marker for poor clinical outcomes in patients with HCC. In addition to showing the oncogenic role of GBA in HCC cell malignancy, we also revealed by both gain- and loss-of-function experiments that GBA dysregulation led to impairment of the autophagic degradation process, consistent with the findings based on the clinical HCC tissues and the orthotopic mouse model of HCC in vivo (Fig. 8). These novel mechanisms ensure the anti-HCC potential of drugs that target GBA. Interestingly, we identified GBA as one of the direct targets of ART through integrating probe labeling and, pulldown assays and SPR analysis. Subsequent pharmacological experiments demonstrated that ART induced autophagy initiation in human HCC cells and orthotopic HCC tissues, as evidenced by the enhanced protein expression level of LC3B, increased number of GFP-LC3 puncta, and induced formation of double-membrane vacuoles. However, ART treatment caused a marked increase in p62 expression. Addition of the late-stage autophagy inhibitor BAF further enhanced LC3B and p62 expression and increased autophagosome accumulation, suggesting that ART blocks autophagic flux in HCC cells. Mechanistically, we found that the ART-induced impairment of autophagic degradation may result from reduced GBA expression (Fig. 8). Cell viability assays and flow cytometric analysis indicated that ART-induced autophagosome accumulation was responsible for ART-induced apoptosis in HCC cells, and this hypothesis was supported by the observation that the suppression of autophagy at the late stage by BAF enhanced the anti-HCC efficacy of ART in vitro and in vivo. These findings identified a novel oncogenic and autophagic machinery component, GBA, as a therapeutic target in HCC and revealed a new pharmacological mechanism by which ART suppresses HCC through targeting of GBA, implying the anti-HCC potential of ART as a novel lysosomal autophagy inhibitor.

Overwhelming evidence suggests that the lysosomal hydrolytic enzyme GBA may play essential roles in the autophagy–lysosome pathway in both macroautophagy and chaperone-mediated autophagy^32^. Our data indicate that high GBA expression accompanied by active autophagic flux was associated with advanced tumor progression and unfavorable prognosis in HCC patients. Mechanistically, the reduced expression of GBA led to suppression of cell proliferation, colony formation and cell cycle progression and induced apoptosis in human HCC cells; these effects were reversed by the GBA activator. In addition, knockdown of GBA expression impaired autophagic degradation and led to autophagosome accumulation via increased conversion of LC3A to LC3B and upregulation of SQSTM1/p62 protein expression in vitro and in vivo. Interestingly, the suppressive effects of GBA knockdown on cancer cell malignancy were partially dependent on the inhibition of autophagy.

During the screening of candidate therapeutic drugs that target GBA, ART, as a clinical drug with high efficiency, rapidity, low toxicity and reduced drug resistance, attracted our attention. The promising use of ART in the treatment of cancer has been a main focus in past decades. Large bodies of evidence suggest its essential roles in various essential cellular malignant processes, such as viability, motility and apoptosis, and ART is an attractive anticancer agent^33^. Mechanistically, our previous study found that ART-induced lysosome activation synergized with sorafenib-mediated pro-oxidant effects by promoting sequential reactions, including lysosomal cathepsin B/L activation, ferritin degradation, lipid peroxidation, and consequent ferroptosis^34^. Jiang et al.^35^ observed that ART can regulate the labile iron pool and effectively induce reactive oxygen species-dependent cell death in multiple HCC cell lines by promoting lysosomal degradation of the iron storage protein ferritin through lysosome acidification. Jing et al.^36^ revealed a function of ART that can induce HCC cell apoptosis via PI3K/AKT/mTOR pathway inhibition. Ilamathi et al.^37^ also identified ART as a potential anti-HCC agent via inhibition of IL-6-driven STAT-3–DNA binding activity. However, the direct targets of ART influencing its anticancer efficacy are still unclear. Herein, following verification of the direct drug–target interaction between ART and the GBA protein, we demonstrated that ART inhibited HCC cell proliferation by impairing late-stage autophagy and inducing apoptosis by reducing the expression of its target protein GBA. In addition, ART enhanced LC3A-LC3B conversion and SQSTM1/p62 accumulation, as evidenced by the increasing number of LC3B puncta in HCC cells, and these effects were further enhanced by the addition of the late-stage autophagy inhibitor BAF. Conversely, activation of GBA by the specific activator LT1-291 significantly reversed the ability of ART to inhibit HCC cell proliferation and increase the G1 population, LC3B conversion and SQSTM1/p62 accumulation. To improve the success rate of clinical translation, both a rat model of DEN-induced HCC, which specifically simulates the aggressive progression from hepatic fibrosis to HCC, and an orthotopic mouse model of HCC were used here to investigate the pharmacological effects and molecular mechanisms by which ART elicits its anti-HCC effects. All surviving rats in the DEN model group had developed HCC by the end of the experiment. The general and pathological observations, indicated that our DEN-induced HCC model reproduced the inflammation stage, the fibrosis stage and the HCC stage, and all three stages with mixed features were observed from week 12 to week 18. Notably, ART treatment effectively inhibited the development of HCC and preneoplastic lesions and the serum levels of liver function-related parameters and fibrosis and tumor-related markers in rats with DEN-induced injury, implying the potential of ART to suppress the inflammation–fibrosis–cancer axis. Moreover, we validated the direct anticancer effect of ART using an orthotopic mouse model of HCC. As expected, ART treatment effectively suppressed tumor growth. Importantly, we examined its regulatory effects on autophagy and found that the number of autophagosomes was markedly increased in ART-treated tumor samples and was further increased by the GBA activator. ART increased the levels of LC3B and SQSTM1/p62 and reduced the level of GBA in tissues of both DEN-induced and orthotopic HCC, in line with the results of in vitro experiments.

In conclusion, this preclinical study identified the novel oncogenic and autophagic degradation machinery component GBA, which is one of the direct targets of ART. The in vitro and in vivo experiments provide insights into the pharmacological mechanisms by which ART suppresses HCC through regulation of GBA, suggesting the promising use of ART as an adjuvant or a direct autophagy inhibitor for HCC therapy. Furthermore, it is of therapeutic value in HCC to increase the accumulation of autophagosomes with anticancer agents.

## Supplementary information

Supplementary File
