# Supplementary material for: Preclinical investigation of artesunate as a therapeutic agent for hepatocellular carcinoma via impairment of glucosylceramidase-mediated autophagic degradation
Source: Exp Mol Med. 2022 Sep 20;54(9):1536–48. doi: 10.1038/s12276-022-00780-6 (PMC9535011; doi:10.1038/s12276-022-00780-6)

Supplementary File: for "Preclinical Investigation of Artesunate as a Therapeutic Agent for Hepatocellular Carcinoma via Impairment of Glucosylceramidase-Mediated Autophagic Degradation"

Section 1: Human HCC Samples

In our clinical cohort, a total of 99 cancerous and 18 adjacent non-cancerous liver tissue samples from 100 HCC patients were collected at the Fifth Medical Centre of Chinese PLA General Hospital. Among them, three paired human primary HCC tissues and adjacent non-cancerous liver tissues were used to microarray assay, and other tissues were used for the experimental validation of GBA expression and its associations with various clinicopathological characteristics. The eligibility criteria for HCC patient recruitment were as follows: (1) histologically-confirmed HCC; (2) received surgical resection; (3) availability of complete clinical data; (4) none received preoperative chemotherapy or radiotherapy; and (5) no history of other malignancy. Tissue samples were collected and stored at liquid nitrogen immediately after surgery. To evaluate the prognostic value of GBA expression, the publicly available dataset of HCC [Provisional, Tumor Samples with mRNA data (RNA Seq V2) (373 samples)] was collected from The Cancer Genome Atlas (TCGA) with mRNA sequencing expression data and relevant clinical information. The date of the last follow-up examination or the initial diagnosis until death or relapse was used to calculate overall survival and disease-free survival. The clinicopathological characteristics of our clinical cohort and the TCGA cohort, including patients' age, gender, preoperative serum AFP level, tumor stage, tumor grade, cirrhosis and invasion status were summarized in **Supplementary Table 1-3**, respectively.

Section 2: siRNA targeting sequences

The siRNA targeting sequences were as follows: negative control, sense, 5'-UUC UCC GAA CGU GUC ACG UTT-3', antisense, 5'-ACG UGA CAC GUU CGG AGA ATT-3'; si-GBA-313, sense, 5'-CCA CAU ACU GUG ACU CCU UTT-3', antisense, 5'-AAG GAG UCA CAG UAU GUG GTT-3'; si-GBA-1255, sense, 5'-CCA UGC UCU UUG CCU CAG ATT-3', antisense, 5'-UCU GAG GCA AAG AGC AUG GTT-3'; si-GBA-1620, sense, 5'-GCU GUU GUG GUC GUG CUA ATT-3', antisense, 5'-UUA GCA CGA CCA CAA CAG CTT-3' for human GBA.

Section 3: Gene Expression Profiling

Total RNA was isolated from three paired human primary HCC and adjacent non-cancerous liver tissue samples using QIAGEN RNeasy Mini Kit according to the manufacturer’s protocol (Cat.No.217004, Qiagen, Hilden, Germany). RNase-Free DNase Set (Cat. No.79254, Qiagen, Hilden, Germany) was used to remove residual DNA contamination. The concentration of total RNA was measured using a NanoDrop spectrophotometer (Nanodrop technologies, Montchanin, DE, USA). mRNA expression profiles in HCC and adjacent non-cancerous liver tissue samples were detected using Affymetrix Clariom D Human array carried out by Shanghai GMINIX Biotechnology Corporation, Shanghai, China. The data had been deposited in NCBI's Gene Expression Omnibus and are accessible through GEO Series accession number GSE166163 (https://www.ncbi.nlm.nih.gov/geo/query/acc.cgi?acc=GSE166163).

Section 4: Real-time Quantitative PCR Analysis

GBA mRNA expression levels in clinical sample tissues and HCC cells were examined by quantitative PCR using ABI 7900HT quantitative PCR system. GAPDH was used as an internal control for normalization and quantification. All primer sequences are: GBA, forward, 5' -CAT CCG CAC CTA CAC CTA TGC-3', reverse,

5'-TGA GCT TGG TAT CTT CCT CTG G-3'; GAPDH, forward, 5' -GGA GCG AGA TCC CTC CAA AAT-3', reverse, 5' -GGC TGT TGT CAT ACT TCT CAT GG-3'. Relative quantification of GBA mRNA expression was evaluated using the comparative cycle threshold method.

Section 5: Immunohistochemistry & Immunofluorescence Analyses

The immunohistochemical staining was conducted using an UltraSensitive SP (mouse/rabbit) immunohistochemistry kit (KIT-9707, MX Biotechnologies, Fuzhou, China), which contained endogenous peroxidase blocking solution, serum, secondary antibody, streptavidin-peroxidase, and diaminobenzidine (DAB) substrate-chromogen (DAB-0031, MX Biotechnologies, Fuzhou, China). The primary antibodies to GBA (1:100, Ab128879, ABCAM, UK) and p62 (1:200, BA2849, BOSTER, Wuhan, China) were used. Immunofluorescence studies were carried out in formalin-fixed paraffin samples. The antibodies were 6-diamidino-2-phenylindole (DAPI, 1:1,000, Invitrogen, USA), anti-LC3 (1:200, 12741, Cell Signaling Technology, USA) and anti-Cy3 (1:200, GB21403, Servicebio, Wuhan, China). Autophagy was quantified by calculating the average number of LC3B puncta per cell in at least five high-power fields.

Section 6: Cell Proliferation, Cell Cycle, Cell Apoptosis & Cell Colony Assays

For cell proliferation assay, cell viabilities of HepG2 and MHCC-97H cells in different groups was detected with a CCK-8 assay (C0039, Beyotime Biotechnology, Jiangsu, China). For cell cycle assay, HepG2 and MHCC-97H cells were respectively seeded into six-well plates and reached approximately 70%-80% confluence. Cells were collected and fixed in 70% ethanol at 20 ºC overnight. After resuspending the cells, 20 mg/mL PBS, 200 mg/mL RNaseA, and 0.1% Triton X-100 were added into the solution. Then, the cell suspension was incubated in the dark for 30 min at 4ºC. The cell cycle assay was analyzed by flow cytometry (NovoCyte, Agilent, Tokyo, Japan). Cell apoptosis assay was performed using an ANXA5/annexin V-FITC Apoptosis

Detection Kit (BB-4101-2, BestBio) following the manufacturers' instructions and analyzed with flow cytometry. For colony formation assay, both HepG2 and MHCC-97H cells were inoculated into six-well culture plate with 500 cells/well and cultured for 10 d before fixed with 4% paraformaldehyde and stained with crystal violet (C3886, Sigma, Germany).

Section 7: Antibodies

The detailed information of the primary antibodies is provided as follows: anti-GBA (1:100, Ab128879, ABCAM, UK), anti-SQSTM1/p62 (1:200, BA2849, BOSTER, Wuhan, China), anti-LC3 (1:200, 12741, Cell Signaling Technology, USA) and anti-GAPDH (1:100, 10494-1-AP, Proteintech, USA).

Section 8: Pull down/LC-MS/MS and targets validation

To identify the interacting cellular targets of ART, pull-down (PD) experiments were carried out, and followed by Western blotting and LC-MS/MS. HepG2 cells were treated with ART-probe in the presence or absence of ART (DMSO never exceeded 1% in the final solution) at a final concentration of 20 μM. After 2 h of incubation, the medium was aspirated, and cells were washed twice with PBS to remove excessive probe. The cells were lysed with RIPA lysis buffer and centrifuged for 10 min (14000 rpm, 4 °C) to get a soluble protein solution. Eventually, the protein concentrations were determined by BCA protein assay and then diluted to 1 mg/mL with PBS. A freshly premixed click chemistry reaction cocktail was added (50 μM Biotin-N3from 30 mM stock solution in DMSO, 100 μM TBTA from 100 mM freshly prepared stock solution in DMSO, 1 mM TCEP from 1 M freshly prepared stock solution in deionized water, and 1 mM $CuSO_4$ from 1 M freshly prepared stock solution in deionized water). The reaction was further incubated for 1 h with gentle mixing prior to precipitation by addition of pre-chilled acetone (-20 °C). Precipitated proteins were subsequently collected by centrifugation (13000 rpm × 10

min at 4 °C) and dissolved in PBS containing 1% SDS. Upon incubation with streptavidin beads for 2 hours at r.t., the beads were washed with PBS containing 0.5% SDS (3 × 1 mL) and PBS (3 × 1 mL). The enriched proteins was eluted by 2 × loading buffer at 95 °C for 10 min and separated by SDS-PAGE (10%). Control pull-down experiments using the DMSO. Western blotting experiments were carried out using the corresponding antibodies (Abcam, UK, ab128879).

For on beads digestion, beads were resuspended in 500 μL 6 M urea in PBS, DTT was added at a final concentration of 10 mM, and the reaction was incubated for 37 ºC for 30 min. For alkylation, IAA was added at a final concentration of 25 mM and incubated for 30 min at r.t. in dark. For the digestion, 150 μL 2 M urea in PBS, 1 mM CaCl2 in 50 mM NH4HCO$_3$ and 2 μL of trypsin (0.5 μg /μL) were added. The reaction was incubated at 37 ºC overnight. The reaction was quenched by adding 2 μL of TFA, then the resulting peptide supernatant was transferred to a new tube. Upon extraction, the samples were desalted by C18 column and then submitted for MS analysis.

The peptides were separated and analyzed on Thermo-Fisher Orbitrap Fusion lumos. About 1 μg of peptides were separated in an home-made column (75 μm x 15 cm) packed with C18 AQ (5 μm, 300Å, MichromBioResources, Auburn, CA, USA) at a flow rate of 300 nL/min. Mobile phase A (0.1% formic acid in 2% ACN) and mobile phase B (0.1% formic acid in 98% ACN) were used to establish a 60 min gradient comprised of 2 min of 5% B, 40 min of 5-26% B, 5 min of 26-30% B, 1 min of 30-35% B, 2 min of 35-90% B and 10 min of 90% B. Peptides were then ionized by electrospray at 1.9 kV. A full MS spectrum (375-1400 m/z range) was acquired at a resolution of 120,000 at m/z 200 and a maximum ion accumulation time of 20 ms. Dynamic exclusion was set to 30 s. Resolution for HCD MS/MS spectra was set to 30,000 at m/z 200. The AGC setting of MS and MS2 were set at 3E6 and 1E5, respectively. The 20 most intense ions above a 1.0E3 counts threshold were selected for fragmentation by HCD with a maximum ion accumulation time of 60 ms. Isolation width of 1.6 m/z units was used for MS2. Single and unassigned charged ions were excluded from MS/MS. For HCD, normalized

collision energy was set to 25%.

The raw data were processed and searched with PD 2.4 with MS tolerance of 4.5 ppm, and MS/MS tolerance of 20 ppm. The UniProt human protein database (release on 2020_01) and database for proteomics contaminants were used for database searches. Reversed database searches were used to evaluate false discovery rate (FDR) of peptide and protein identifications. Two missed cleavage sites of trypsin were allowed. Carbamidomethylation (C) was set as a fixed modification, and oxidation (M), Acetyl (Protein N-term) and deamidation (NQ) were set as variable modifications. The FDR of both peptide identification and protein identification is set to be 1%. Data were summarized from three biological replicates.

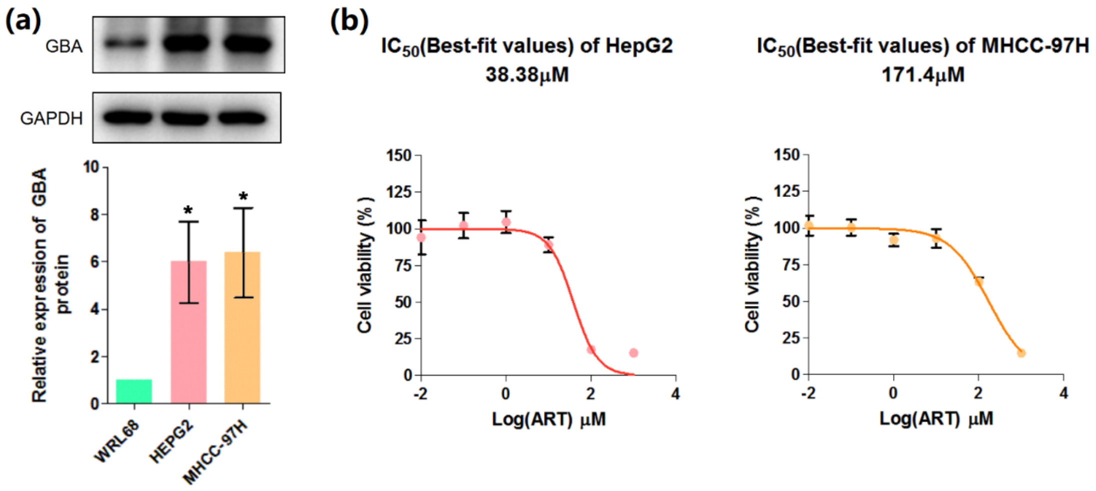

Supplementary Fig.1 (a) Expression level of GBA protein in normal human liver cell (WRL68) and two HCC cell lines (HepG2 and MHCC-97H). (b) Cell viability of HepG2 and MHCC-97H cells treated by various doses of ART.

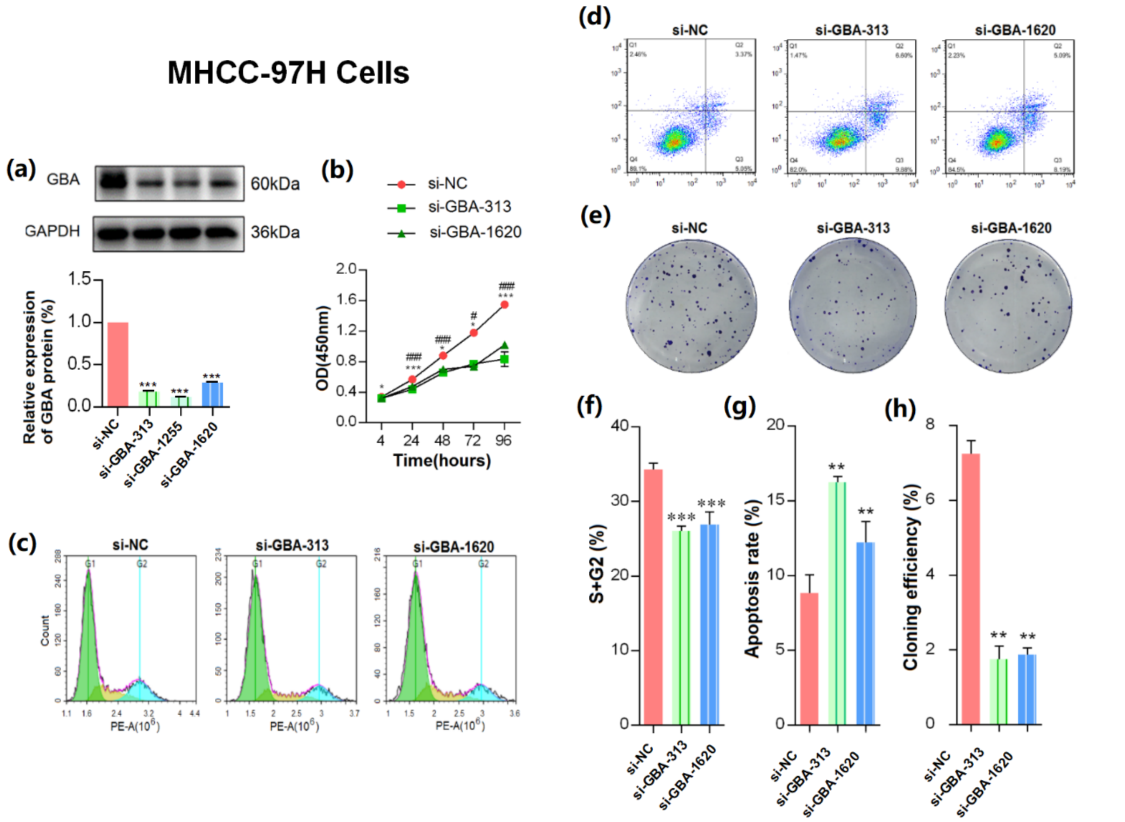

Supplementary Fig.2 Effects of GBA on cellular malignancies of MHCC-97H cells.

(a) Expression levels of GBA protein in MHCC-97H cells transfected with three si-GBA vectors (si-GBA-313, si-GBA-1250 and si-GBA-1620) detected by western blot analysis. Representative column diagrams show results of relative protein expression. (b) Cell viability of MHCC-97H cells transfected with si-GBA-313 and si-GBA-1620 determined by CCK-8 assay. (c) and (f) Flow cytometry, (d) and (g) cell apoptosis, (e) and (h) cell cloning efficiency analysis of the cell cycle distribution of MHCC-97H cells transfected with si-GBA-313 and si-GBA-1620, respectively. Data are expressed as the mean±SD. *$p < 0.05$, **$p < 0.01$, ***$p < 0.001$, si-GBA groups comparison with si-NC group.

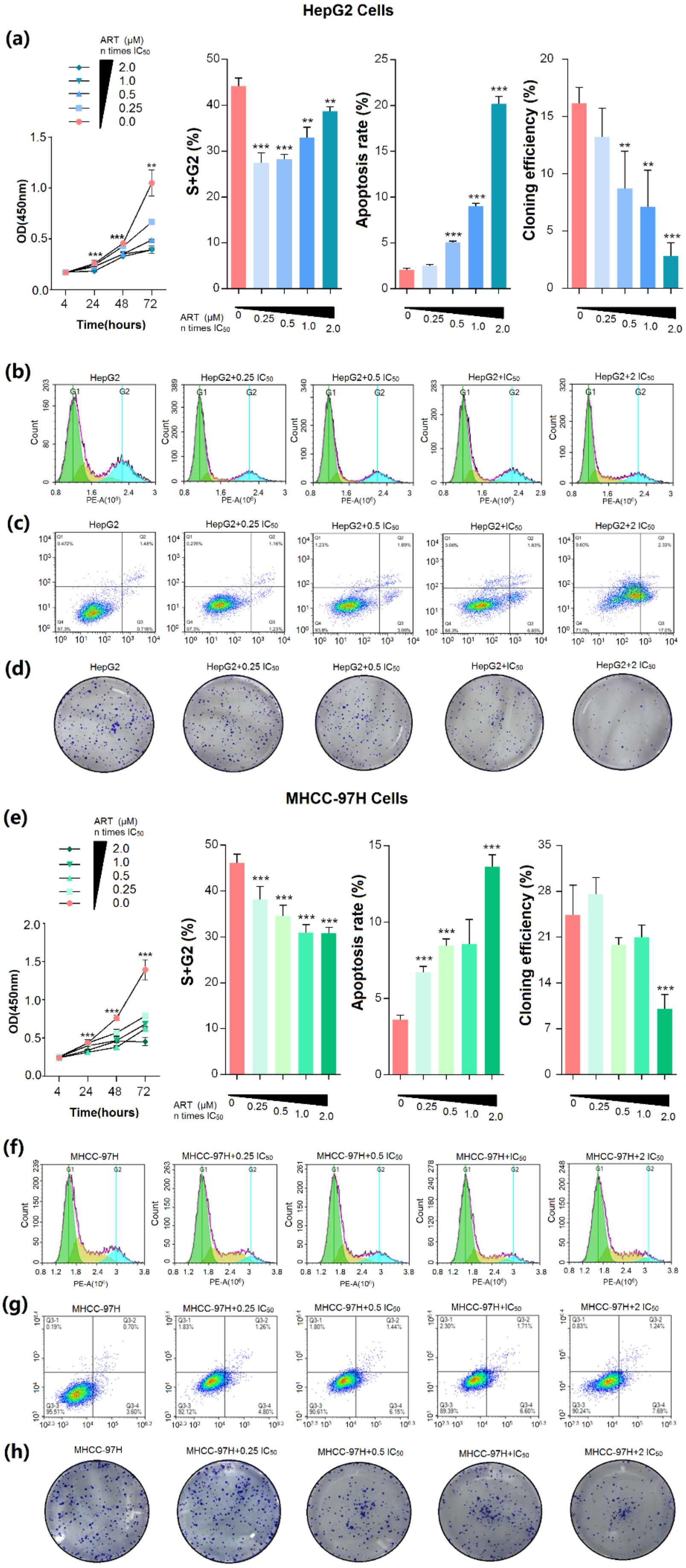
HepG2 Cells
(a)
ART (μM)
n times IC50
2.0
1.0
0.5
0.25
0.0
OD(450nm)
Time(hours)
S+G2 (%)
Apoptosis rate (%)
Cloning efficiency (%)
(b)
HepG2
HepG2+0.25 IC50
HepG2+0.5 IC50
HepG2+IC50
HepG2+2 IC50
Count
PE-A(10⁶)
(c)
(d)
MHCC-97H Cells
(e)
(f)
MHCC-97H
MHCC-97H+0.25 IC50
MHCC-97H+0.5 IC50
MHCC-97H+IC50
MHCC-97H+2 IC50
(g)
(h)

Supplementary Fig.3 Effects of ART on cellular malignancies of HCC cells.

(a) and (e) Cell viabilities of HepG2 and MHCC-97H cells treated with ART in different dosages determined by CCK-8 assay. (b) and (f) Cell cycle of HepG2 and MHCC-97H cells treated with ART in different dosages determined by flow cytometry.

(c) and (g) Cell apoptosis of HepG2 and MHCC-97H cells treated with ART in different dosages. (d) and (h) Cell cloning efficiency analysis of the cell cycle distribution of HepG2 and MHCC-97H cells treated with ART in different dosages. Data are expressed as the mean±SD. *$p < 0.05$, **$p < 0.01$, ***$p < 0.001$, ART treatment groups comparison with Blank control group.

(a)

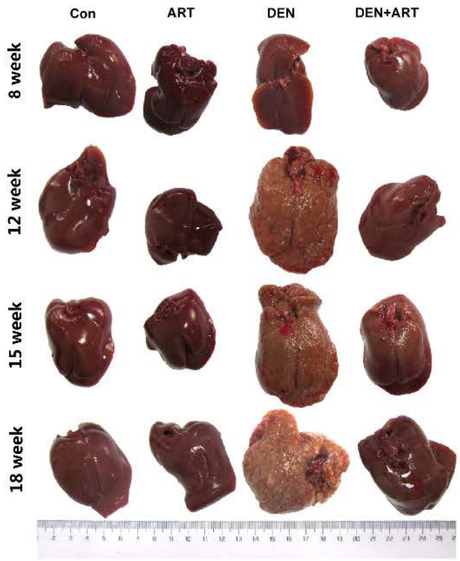

(b)

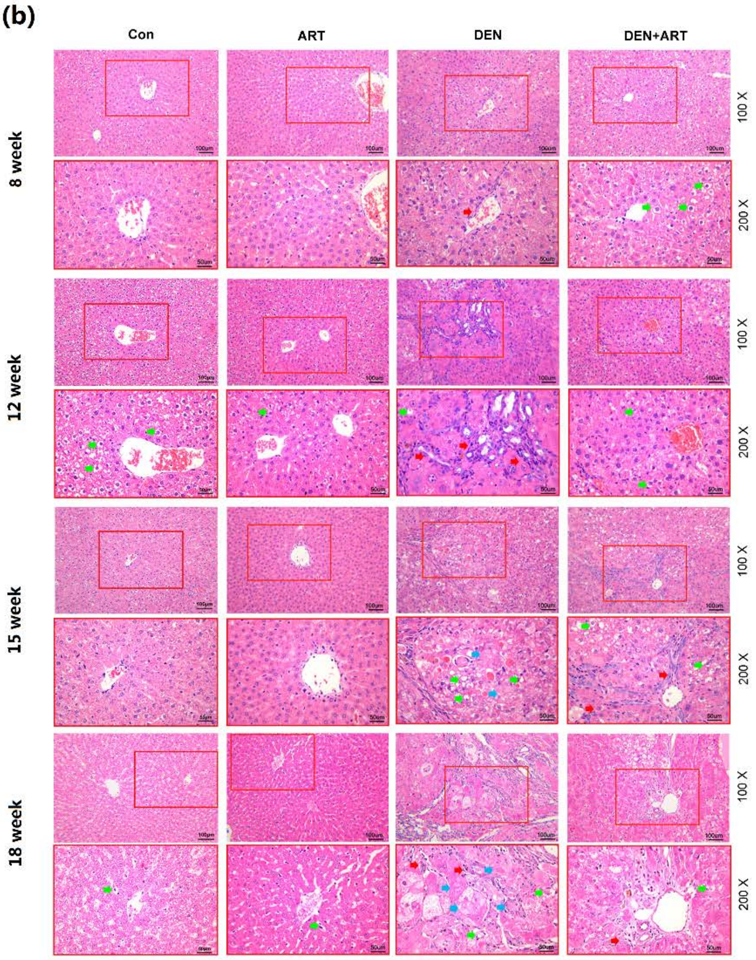

Supplementary Fig.4 (a) Effects of ART on the generation of neoplastic lesions and associated fibrosis on DEN-induced HCC rats, as well as its influence on

the body weight and the liver index. (b) Effects of ART on the histological changes of liver in DEN-induced HCC rats.

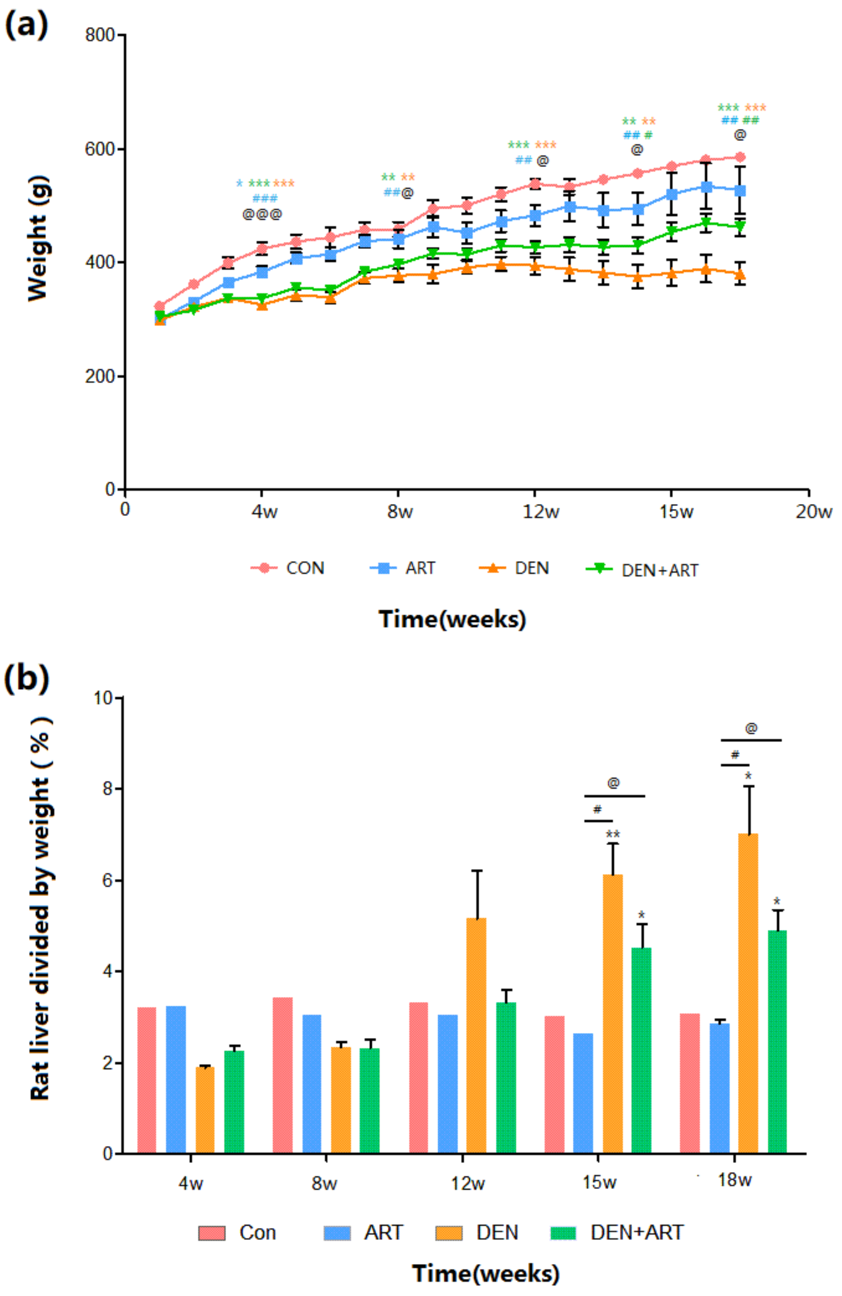

Supplementary Fig.5 (a) The body weight of rats in different groups. (b) The liver index of rats in different groups.

Data are expressed as the mean±S.D. '#', '##', and '###', P<0.05, P<0.01, and P<0.001, respectively, comparison with the normal group. '@', '@@', and '@@@', P<0.05, P<0.01, and P<0.001, respectively, comparison with the Normal+ART group.

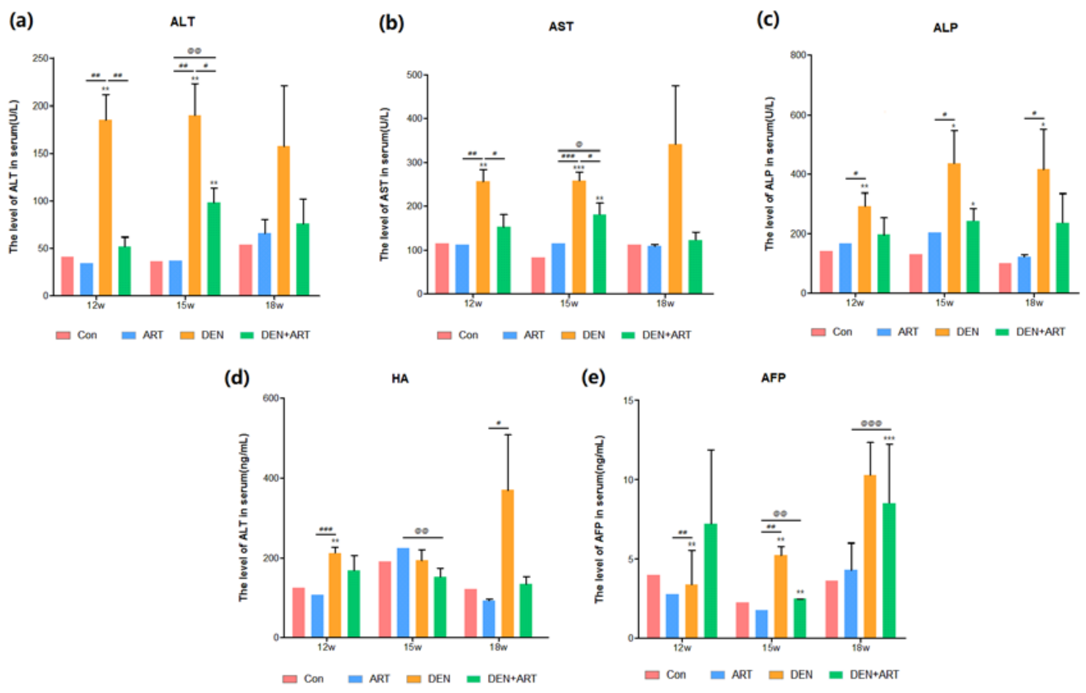

Supplementary Fig.6 Effects of ART on the liver function-related serum parameters (ALT, AST, ALP), as well as fibrosis (HA) and tumor-related serum markers (AFP).

(a-e) The serum levels of ALT, AST, ALP, HA and AFP in different groups. Data are expressed as the mean±S.D. ‘#’, ‘##’, and ‘###’, P<0.05, P<0.01, and P<0.001, respectively, comparison with the normal group. ‘@’, ‘@@’, and ‘@@@’, P<0.05, P<0.01, and P<0.001, respectively, comparison with the normal+ART group. ‘*’, ‘**’, and ‘***’, P<0.05, P<0.01, and P<0.001, respectively, comparison with the DEN group.

Supplementary Table 1 Associations between GBA expression and various clinicopathological features of HCC patients in our clinical cohort

Clinicopathological Features		No. of Patients	GBA expression		*P*
			High（n,%）	Low（n,%）	
**Gender**	Male	88	47 (53.41)	41 (46.59)	>0.05
	Female	11	3 (27.27)	8 (72.73)	
**Age (years)**	<52	45	25 (55.56)	20 (44.44)	>0.05
	≧52	54	25 (46.30)	29 (53.70)	
**Preoperative serum AFP level (Positive: higher than 400)**	Positive	33	22 (66.67)	11 (33.33)	0.01<p<0.05
	Negative	66	28 (42.42)	38 (57.58)	
**Tumor stage**	T2	84	44 (52.38)	40 (47.62)	>0.05
	T3	8	4 (50.00)	4 (50.00)	
**Tumor grade**	G2	77	35 (45.45)	42 (54.55)	<0.01
	G3	17	14 (82.35)	3 (17.65)	
**Cirrhosis**	Positive	85	44 (51.76)	41 (48.24)	>0.05
	Negative	14	6 (42.86)	8 (57.14)	
**Invasion**	Positive	81	41 (50.62)	40 (49.38)	>0.05
	Negative	18	9 (50.00)	9 (50.00)	

Supplementary Table 2 Associations between GBA expression and various clinicopathological features of HCC patients in the TCGA dataset

Features		No. of Patients	GBA expression High (n,%)	GBA expression Low (n,%)	P
**Gender**	Male	252	138 (54.76)	114 (45.24)	<0.01
	Female	121	49 (40.50)	72 (59.50)	
**Age (years)**	<61	177	80 (45.20)	97 (54.80)	0.02
	≧61	195	106 (54.36)	89 (45.64)	
**Preoperative serum AFP level**	Positive	149	81(54.36)	68(45.64)	0.08
	Negative	130	66(50.77)	64(49.23)	
**Tumor Stage (American Joint Committee on Cancer)**	T1	182	87 (47.80)	95 (52.20)	>0.05
	T2	95	55 (57.89)	40 (42.11)	
	T3	80	36(45.00)	44(55.00)	
	T4	13	8(61.54)	5(38.46)	
**Metastasis stage (American Joint Committee on Cancer)**	M0	267	135(50.56)	132 (49.44)	0.01<p<0.05
	M1	4	4 (100.00)	0 (0)	
**Disease Free Status**	Non- Recurred	145	79(54.48)	66(45.52)	0.07
	Recurred	176	89(50.57)	87(49.43)	
**Overall Survival**	Living	242	112(46.28)	130(53.72)	0.01

**Status**	Deceased	130	74(56.92)	56(43.08)	
**Liver fibrosis ishak score**	0 (No Fibrosis)	75	36(48.00)	39(52.00)	>0.05
	1～2 （Portal Fibrosis）	31	20(64.52)	11(35.48)	
	3～4 （Fibrous Speta）	28	10(35.71)	18(64.29)	
	5 （Nodular Formation and Incomplete Cirrhosis）	9	6(66.67)	3(33.33)	
	6 （Established Cirrhosis）	70	39(55.71)	31(44.29)	
**Neoplasm Disease Stage (American Joint Committee on Cancer)**	Stage I	172	82(47.67)	90(52.33)	>0.05
	Stage II	86	50(58.14)	36(41.86)	
	Stage III	85	36(42.35)	49(57.65)	
	Stage IV	5	3(60.00)	2(40.00)	
**Neoplasm Histologic Grade**	G1	55	22(40.00)	33(60.00)	>0.05
	G2	178	93(52.25)	85(47.75)	
	G3	122	65(53.28)	57(46.72)	
	G4	12	5(41.67)	7(58.33)	
**Vascular Invasion**	Absent	207	98(47.34)	109(52.66)	0.04
	Present	109	60(55.05)	49(22.95)	

Supplementary Table 3 Clinicopathological features of the discovery and validation cohorts enrolled in the current study

Features		No. of Patients	Discovery Cohort （n=3）	Validation Cohort （n=100）
**Gender**	Male	92	3	89
	Female	11	0	11
**Age (years)**	<52	49	3	46
	≧52	54	0	54
**Preoperative serum AFP level (Positive: higher than 400)**	Positive	34	1	33
	Negative	69	2	67
**Tumor stage**	T2	88	3	85
	T3	8	0	8
	Unknown	7	0	7
**Tumor grade**	G2	62	0	62
	G3	17	2	15
	Unknown	24	1	23
**Cirrhosis**	Positive	89	3	86
	Negative	14	0	14
**Invasion**	Positive	82	0	82
	Negative	21	3	18

Fig. 2a GBA protein expression levels in HepG2 cells transfected with si-GBA-313 and si-GBA-1620 were determined using western blotting.

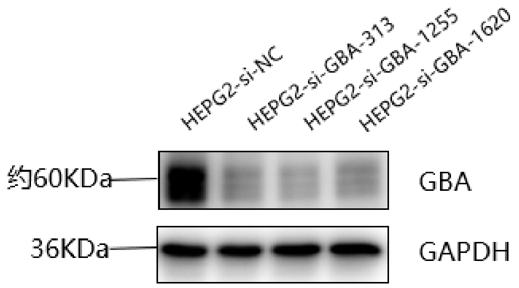

1.GBA

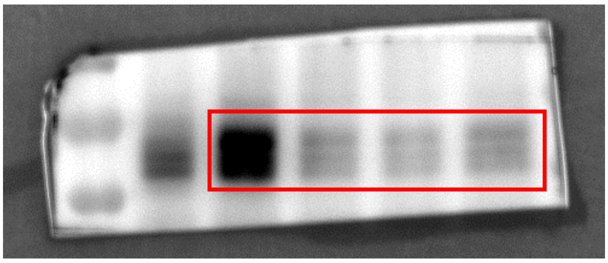

2.GAPDH

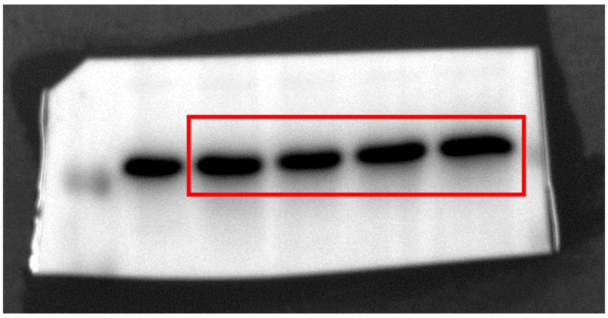

Fig. 2d The protein levels of GBA, p62 and LC3B in different groups of cells were analyzed by western blotting. GAPDH was used as an internal control.

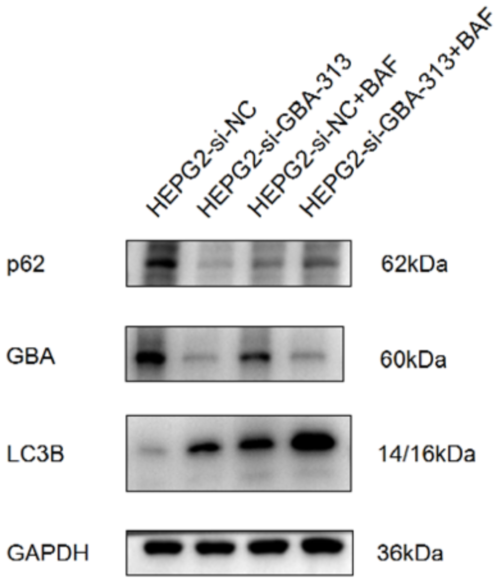

1.GAPDH

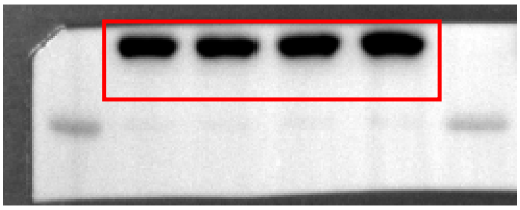

2.p62

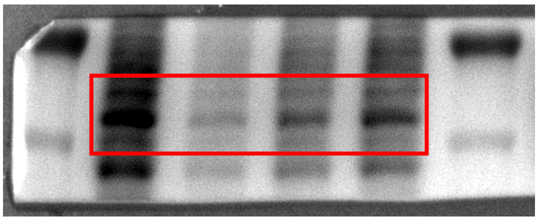

3.GBA

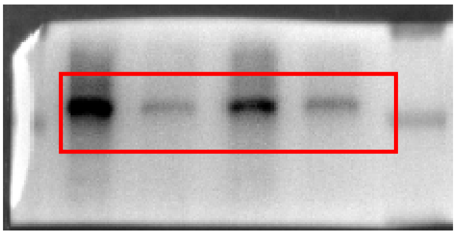

4.LC3B

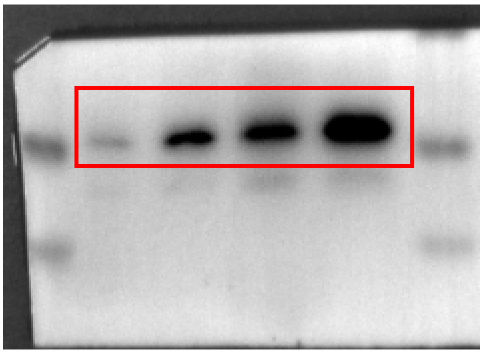

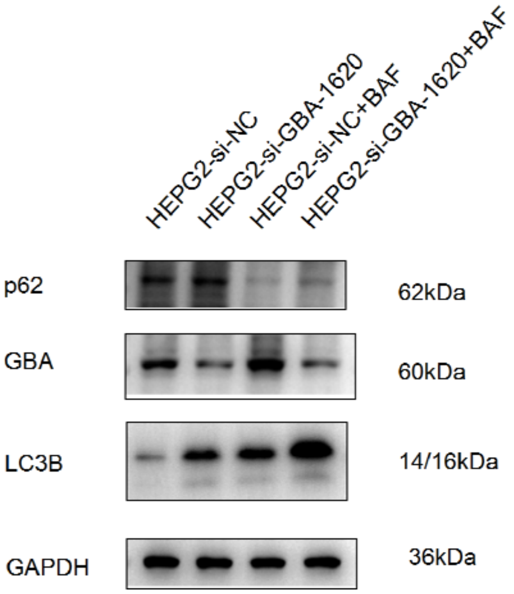

1.GAPDH

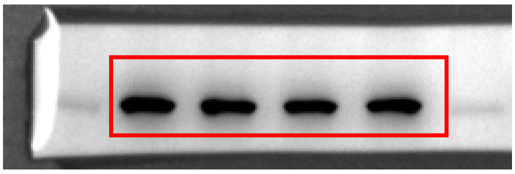

2.p62

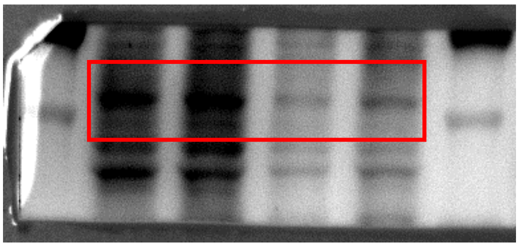

3.GBA

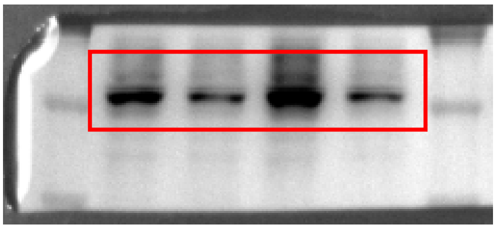

4.LC3B

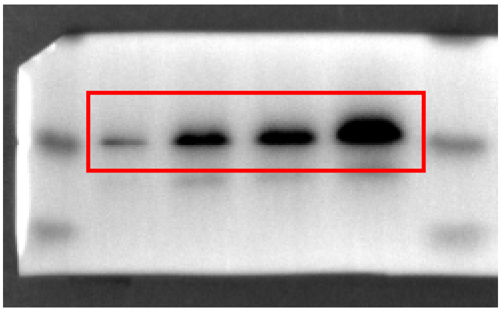

Fig. 3c Pull-down/WB results for target validation of ART.

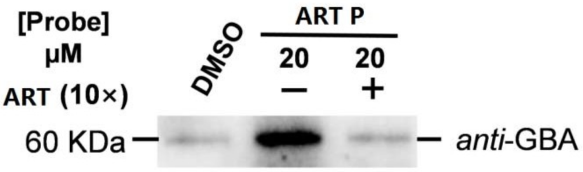

Fig. Pull-down/WB results for target validation of **ART P**

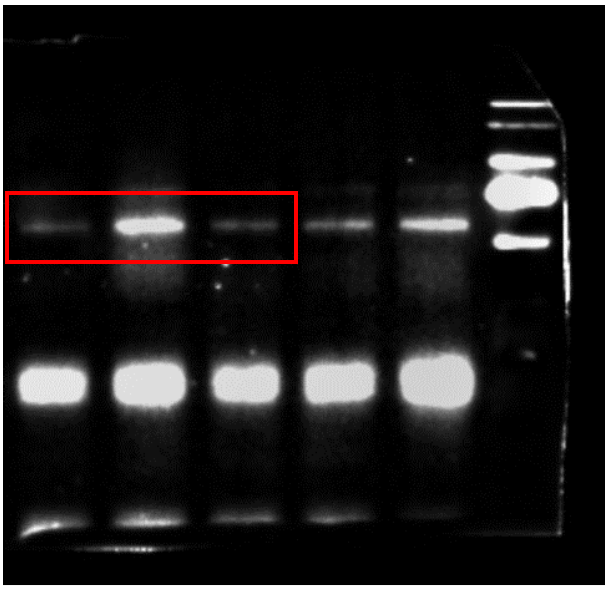

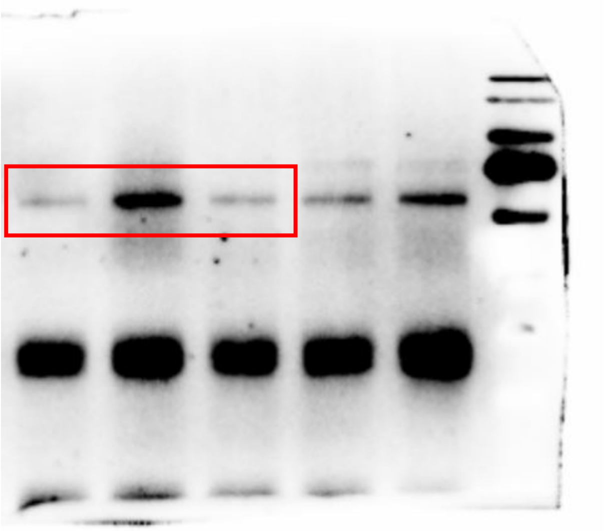

Fig. 4c The effect of ART on GBA, p62 and LC3B protein expressions in different groups of cells were analyzed by western blotting. GAPDH was used as an internal control.

1.GAPDH

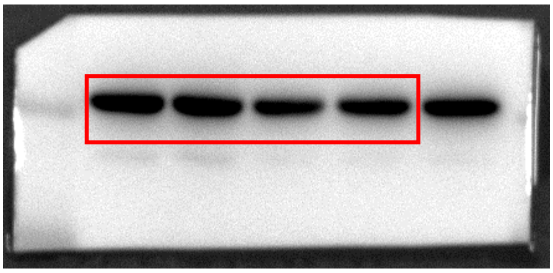

2.P62

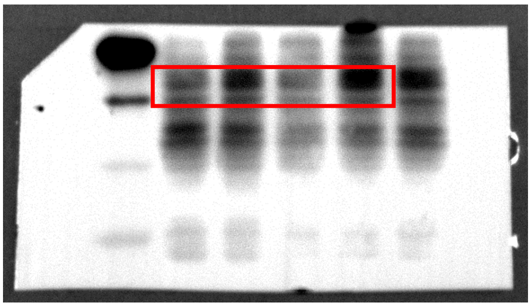

3.GBA

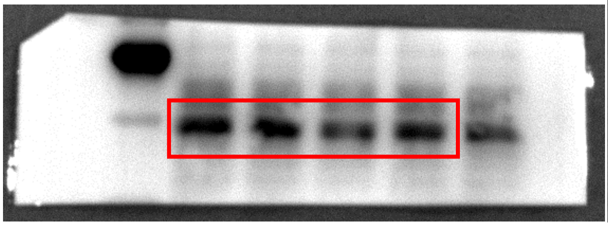

4.LC3B

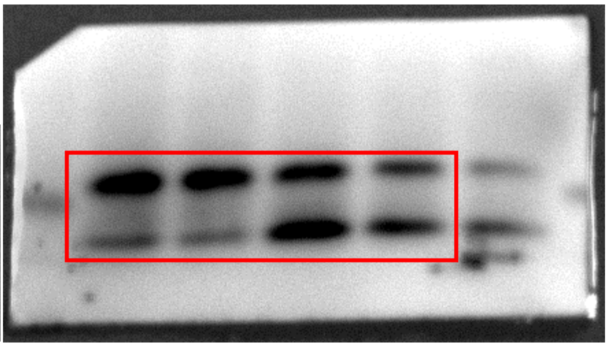

Supplementary Fig. 2a GBA protein expression levels in MHCC-97H cells transfected with si-GBA-313 and si-GBA-1620 were determined using western blotting.

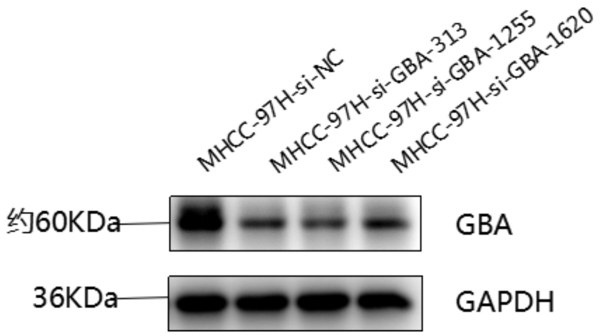

1.GBA

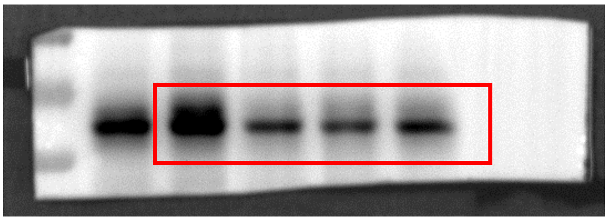

2.GAPDH

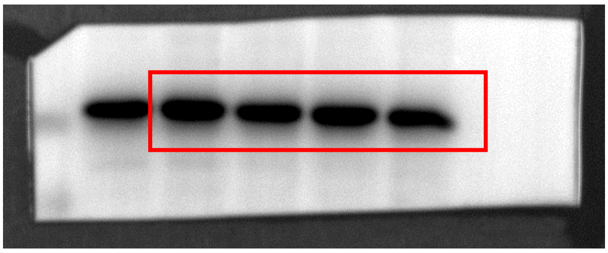

Supplement: Supplementary file 1 — Supplementary File [file 12276_2022_780_MOESM1_ESM.pdf]
